# Three approaches to facilitate invariant neurons and generalization to out-of-distribution orientations and illuminations

**DOI:** 10.1016/j.neunet.2022.07.026

**Published:** 2022-07-30

**Authors:** Akira Sakai, Taro Sunagawa, Spandan Madan, Kanata Suzuki, Takashi Katoh, Hiromichi Kobashi, Hanspeter Pfister, Pawan Sinha, Xavier Boix, Tomotake Sasaki

**Affiliations:** a Artificial Intelligence Laboratory, Fujitsu Limited, 4-1-1 Kamikodanaka, Nakahara-Ku, Kawasaki, Kanagawa 211-8588, Japan; b School of Engineering and Applied Sciences, Harvard University, 29 Oxford Street, Cambridge, MA 02138, USA; c Department of Brain and Cognitive Sciences, Massachusetts Institute of Technology, 77 Massachusetts Avenue, Cambridge, MA 02139, USA; d Center for Brains, Minds and Machines, 77 Massachusetts Avenue, Cambridge, MA 02139, USA

**Keywords:** Out-of-distribution generalization, Object recognition in novel conditions, Neural invariance, Neural selectivity, Neural activity analysis

## Abstract

The training data distribution is often biased towards objects in certain orientations and illumination conditions. While humans have a remarkable capability of recognizing objects in out-of-distribution (OoD) orientations and illuminations, Deep Neural Networks (DNNs) severely suffer in this case, even when large amounts of training examples are available. Neurons that are invariant to orientations and illuminations have been proposed as a neural mechanism that could facilitate OoD generalization, but it is unclear how to encourage the emergence of such invariant neurons. In this paper, we investigate three different approaches that lead to the emergence of invariant neurons and substantially improve DNNs in recognizing objects in OoD orientations and illuminations. Namely, these approaches are (i) training much longer after convergence of the in-distribution (InD) validation accuracy, *i.e*., latestopping, (ii) tuning the momentum parameter of the batch normalization layers, and (iii) enforcing invariance of the neural activity in an intermediate layer to orientation and illumination conditions. Each of these approaches substantially improves the DNN’s OoD accuracy (more than 20% in some cases). We report results in four datasets: two datasets are modified from the MNIST and iLab datasets, and the other two are novel (one of 3D rendered cars and another of objects taken from various controlled orientations and illumination conditions). These datasets allow to study the effects of different amounts of bias and are challenging as DNNs perform poorly in OoD conditions. Finally, we demonstrate that even though the three approaches focus on different aspects of DNNs, they all tend to lead to the same underlying neural mechanism to enable OoD accuracy gains – individual neurons in the intermediate layers become invariant to OoD orientations and illuminations. We anticipate this study to be a basis for further improvement of deep neural networks’ OoD generalization performance, which is highly demanded to achieve safe and fair AI applications.

## 1. Introduction

The object recognition performance of Deep Neural Networks (DNNs) dramatically degrades when the train and test distributions are not identical due to dataset bias (Torralba & Efros, 2011), *i.e*., when tested in out-of-distribution (OoD) conditions. There is a big gap between DNNs and humans when evaluated in OoD conditions. This issue has been getting much interest in recent years (Beery, Horn, & Perona, 2018; Geirhos et al., 2019; Hendrycks et al., 2021; Recht, Roelofs, Schmidt, & Shankar, 2018, 2019), as it severely compromises the safety and fairness of AI applications.

One of the most prominent factors of dataset bias is that objects may appear in a constrained range of orientation and illumination conditions (Alcorn et al., 2019; Barbu et al., 2019). While generalization to OoD orientations and illumination conditions has been long studied in both biological and artificial neural networks, *e.g*., Anselmi, Rosasco, and Poggio (2016), Sinha and Poggio (1996) and Ullman (1996), the computational mechanisms that facilitate such generalization remain as a key outstanding question. Recently, Madan et al. (2022) and Zaidi et al. (2020) have shown that DNNs are capable to overcome bias by transferring the generalization ability obtained from objects seen in a richer set of conditions to the objects seen in biased conditions. Also, the emergence of representations at the individual neuron level in the intermediate layers of the DNN that are selective to categories and invariant to the OoD conditions has been identified as a mechanism that may facilitate such OoD generalization. Invariant neural representations have been studied during decades, *e.g*., Anselmi et al. (2016), and here they appear as the mechanism that allows OoD generalization. This begs the question whether we can further encourage the emergence of invariant neural representations in DNNs in order to further improve OoD generalization.

**Fig. 1.**
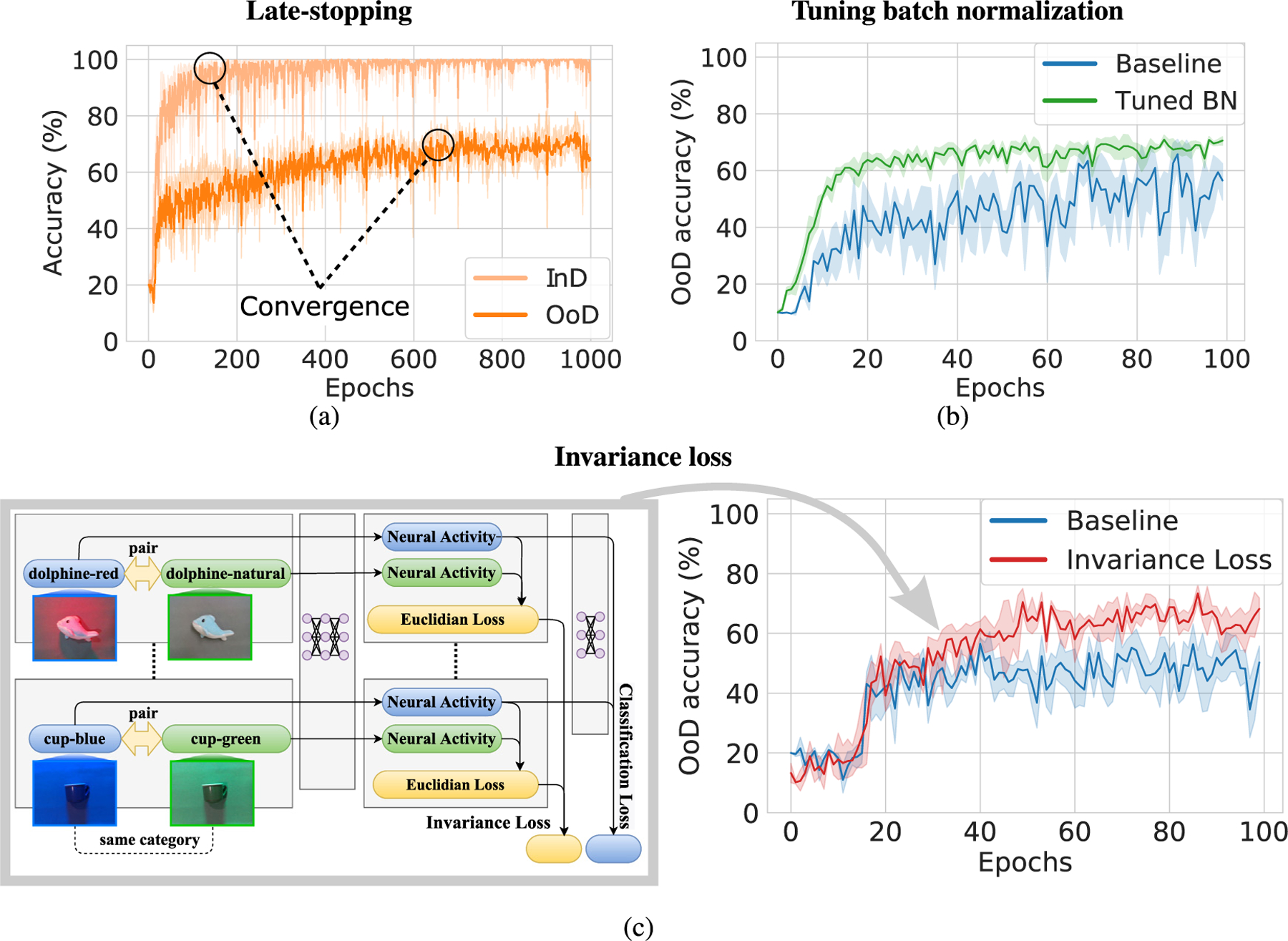
Three approaches to facilitate generalization to objects in out-of-distribution (OoD) orientations and illuminations. (a) Learning curves of in-distribution (InD) test accuracy and OoD accuracy for late-stopping applied to the MiscGoods-illuminations dataset (medium InD data diversity). OoD accuracy converges much later than InD accuracy. (b) Learning curves of the OoD accuracy with and without tuning batch normalization momentum (tuned BN) in the CarsCG-Orientations, dataset (medium InD data diversity). It can be seen that tuning the momentum reduces the oscillation of the OoD accuracy and improves the performance. (c) Left: Conceptual diagram of the invariance loss. Pairs of images that belong to the same category are fed into the DNN. The invariance loss is based on the Euclidean distance between the pairs of the last ReLU activity. The classification loss is calculated with the network output as usual. The total loss is the weighted sum of the invariance and classification losses. Right: Learning curve of OoD accuracy in MiscGoods-Illuminations dataset (medium InD data diversity) when the invariance loss is applied. The OoD accuracy increases by about 20% compared to the baseline. The solid lines in the plots are the mean value. The lighter semitransparent colors surrounding the solid lines indicate 95% confidence interval.

In this paper, we investigate factors that drive the emergence of invariant neurons and as a result substantially boost the DNN ability to recognize objects in OoD orientations and illuminations. In particular, we discover that the following factors, summarized in Fig. 1, have a remarkable impact:

1. *Late-stopping*: DNNs are usually trained until the validation recognition accuracy (which is in-distribution) converges. We found that in many cases the OoD recognition accuracy improves slowly, yet consistently, after the validation (indistribution) accuracy has converged. This finding is surprising as classic machine learning theory suggests early-stopping as a regularization mechanism (Yao, Rosasco, & Caponnetto, 2007b), and we found that the opposite is beneficial to improve OoD generalization in DNNs. We call this approach “late-stopping”.
2. *Tuning the batch normalization parameter:* Batch normalization (BN) is known to have an impact in OoD recognition accuracy (Schneider et al., 2020). We found that tuning the only hyperparameter of BN, *i.e*., the momentum, yields substantial gains of OoD recognition accuracy. This approach is denoted as “tuned BN”.
3. *Neural activity invariance loss:* Motivated by the aforementioned finding in previous works that invariant neural representations leads to improvements of the OoD recognition accuracy, we include an additional term in the loss function to encourage this phenomenon. This loss term takes the Euclidean distance between neural activity corresponding to pairs of images from the same category on an intermediate layer. By minimizing this loss term, the neural activity tends to be invariant for objects of the same category even in different viewing conditions. We do not consider that pairs of images from different categories should have distinguishable neural activity, since the classification loss term already encourages this. We call this approach “invariance loss” in short.

Our results demonstrate that each of these three approaches alone leads to substantial improvements of object recognition in OoD orientations and illumination conditions. Results also corroborate that when any of the three approaches leads to an increase of invariance at the individual neuron level, OoD recognition accuracy improves in the majority of trials.

Experiments are performed in four challenging benchmarks, namely modifications of the MNIST dataset (LeCun, Bottou, Bengio, & Haffner, 1998) and iLab dataset (Borji, Izadi, & Itti, 2016) and two novel datasets we introduce, which are the CarsCG and the MiscGoods datasets. CarsCG contains 3D rendered cars from different orientations, and the MiscGoods dataset consists of images of objects taken with a robotic arm from different viewpoints and controlled illumination conditions. These datasets allow to evaluate the DNN generalization ability to recognize objects in OoD orientations and illumination conditions. Also, they allow to analyze the effects of different amounts of bias and are challenging as DNNs perform poorly in OoD conditions.

**Fig. 2.**
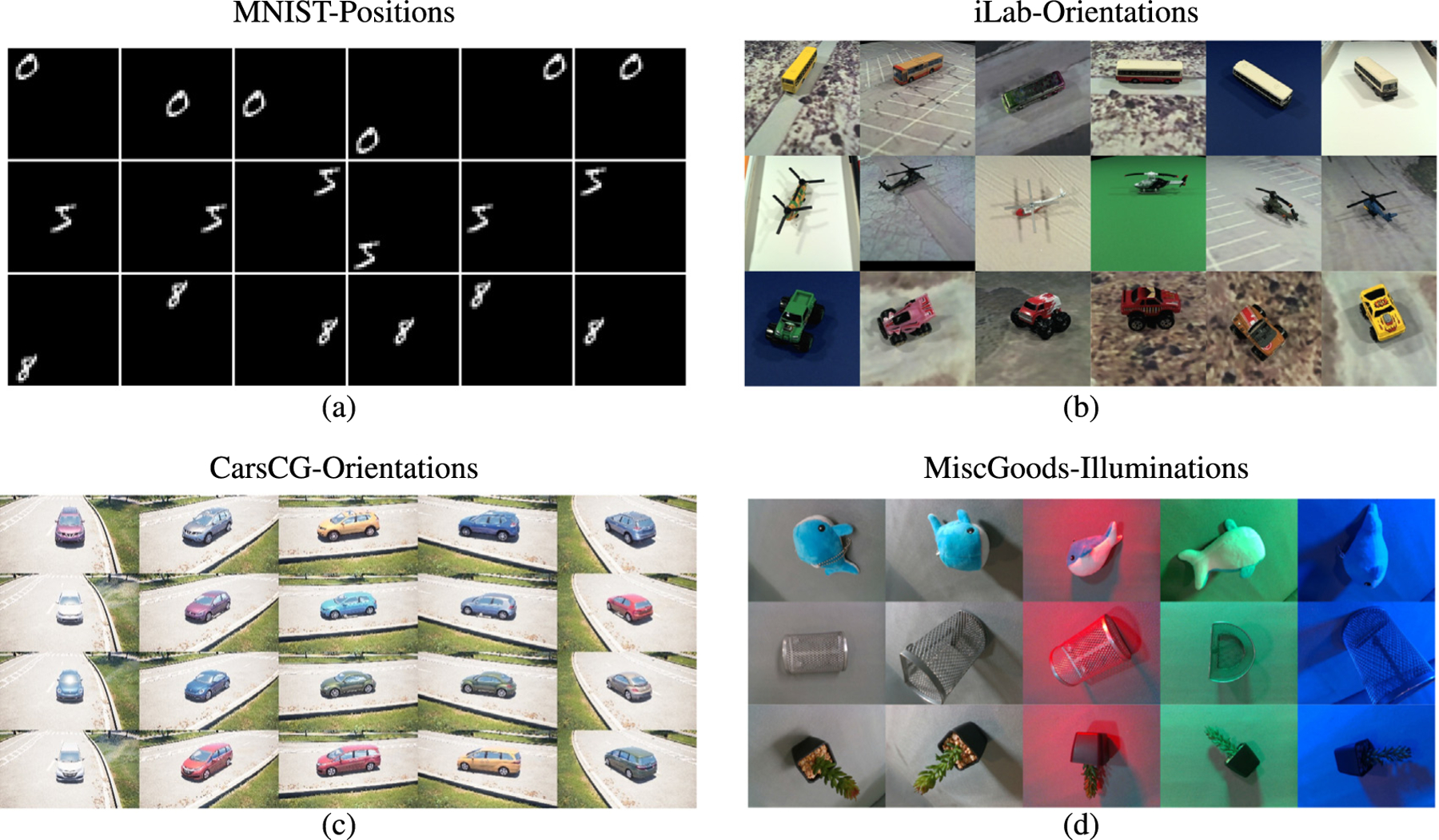
Sample images from four datasets. (a) MNIST-Positions, (b) iLab-Orientations, (c) CarsCG-Orientations, and (d) MiscGoods-Illuminations are shown in each subfigure. Samples from each dataset are arranged in a grid pattern. Each row indicates categories and each column indicates either an orientation or an illumination condition.

*Summary of contributions*. In the following, we summarize the three main contributions of this paper:

1. Three different approaches that substantially improve generalization to OoD viewpoints and illuminations, summarized in Fig. 1.
2. Two new challenging datasets (CarsCG and the MiscGoods) that facilitate the study of OoD orientations and illuminations, displayed in Figs. 2(c) and 2(d).
3. Striking evidence that encouraging the emergence of invariant neurons leads to improvements of object recognition in OoD orientations and illuminations (Fig. 6 and Table 2).

## 2. Previous works

We now review related works to overcoming dataset bias in terms of orientations and illuminations. First, we review works that have the same goal as our work, *i.e*., overcoming biased orientations and illuminations, and then, we review works in other areas of OoD generalization.

### 2.1. OoD orientations and illuminations

Our results add to the growing body of literature to improve the generalization ability of DNNs to OoD orientations and illumination conditions. These are fundamental aspects at the core of object recognition, that are present in all object recognition tasks. Prior efforts leverage synthesized sources of training data (Cubuk, Zoph, Shlens, & Le, 2019; Halder, Lalonde, & Charette, 2019; Kim, Uddin, & Bae, 2021; Qiao, Zhao, & Peng, 2020), 3D models of objects (Angtian, Kortylewski, & Yuille, 2021), specific characteristics of the target domain (Chidester, Zhou, Do, & Ma, 2019; Qi et al., 2018; Sabour, Frosst, & Hinton, 2017), or sensing approaches such as omnidirectional imaging (Cohen, Geiger, Köhler, & Welling, 2018). These approaches add preconceived components to the DNN that need to be adjusted at hand for new objects and conditions. Here, we focus for the first time on a pure learning-based strategy for OoD orientations and illuminations, which is not constrained to specific objects and conditions and can be automatically adjusted to new datasets.

Our investigation build upon theories of biological neural mechanisms for OoD generalization, namely, neural invariance (Anselmi et al., 2016; Quiroga, Reddy, Kreiman, Koch, & Fried, 2005; Riesenhuber & Poggio, 1997; Rust & DiCarlo, 2010). Recent works have shown that those mechanisms also emerge in artificial neural networks and facilitate OoD generalization (Madan et al., 2022; Zaidi et al., 2020). In this paper, we show that the emergence of invariant neurons can be encouraged during training and this leads to substantial improvements of OoD generalization.

### 2.2. Other aspects of OoD generalization

To the best of our knowledge, this paper is the first to investigate learning-based approaches to overcome bias of object’s orientations and illumination conditions. Yet, there are other strands of research that live in neighboring areas, which investigate generalization to new domains and also, overcoming spurious correlations between image features and categories. These research areas use related techniques and concepts to our work, and in the following we review them.

There is a plethora of works that consists on learning representations in several domains that can be easily transferred to new domains, e.g., Carlucci, D’Innocente, Bucci, Caputo, and Tommasi (2019), Dou, Castro, Kamnitsas, and Glocker (2019), Ghifary, Bastiaan Kleijn, Zhang, and Balduzzi (2015), Guo et al. (2020), Jia, Zhang, Shan, and Chen (2020), Li, Pan, Wang, and Kot (2018), Li et al. (2018), Li, Yang, Song and Hospedales (2018) and Volpi et al. (2018). The problem of domain generalization is similar to the problem overcoming dataset bias in our study in the sense that representations that facilitate generalization to novel conditions should be learned. However, in domain generalization the learner has access to multiple domains during training that can be leveraged for generalization, while in the problem of overcoming dataset bias only one training set is available. Recently, several works in domain generalization (Chattopadhyay, Balaji, & Hoffman, 2020; Ilse, Tomczak, Louizos, & Welling, 2020; Rame, Dancette, & Cord, 2021; Xiao, Shen, Zhen, Shao, & Snoek, 2021) highlighted the need of invariant representations to obtain further improvements in generalization, which further motivates investigating invariance for dataset bias.

Many datasets are biased in a way that a specific image feature consistently appears in images of the same category. DNNs tend to learn that those features are informative of the category (Geirhos et al., 2020). This form of dataset bias is different from the bias in the object orientation and illumination conditions, which do not necessarily lead to spurious correlations. Recently, there have been several works that address spurious correlations. These are based on automatically detecting the features that spuriously correlate with the category, and encourage the DNN not to rely on those features (Arjovsky, Bottou, Gulrajani, & Lopez-Paz, 2019; Sagawa, Koh, Hashimoto, & Liang, 2020). Ahmed, Bengio, van Seijen, and Courville (2021) introduced PGI, which is a method that effectively alleviates the effect of spurious correlation caused by biased object background. This work exploits the assumption that the training distribution also contains examples without spurious correlations. CMMD (Li et al., 2018) is another method, which uses idea of maximum mean discrepancy (MMD). CMMD and PGI employ EIIL (Creager, Jacobsen, & Zemel, 2021) to classify the images of an category with the features that spuriously correlate with the category and without them. Then, invariance is encouraged across these two groups of images. Thus, invariance appears once more as a facilitator of generalization.

Recently, several researchers have pointed out that no single OoD algorithm can achieve high performance for all problem domains (Gulrajani & Lopez-Paz, 2021; Hendrycks et al., 2021; Wiles et al., 2022). It also has been reported that even very simple methods can achieve performance beyond state-of-the-art (Djolonga et al., 2021; Wiles et al., 2022). We show that for orientations and illuminations, which are fundamental aspects of object recognition, the approaches we introduce in this paper are superior to the most successful aforementioned generic methods for OoD generalization. Also, we provide insights about the neural mechanisms that facilitate such improvements.

## 3. Performance degradation on OoD conditions

In this section, we introduce the methodology to evaluate the accuracy of the DNN in OoD conditions. First, we describe the procedure of the bias-controlled experiment. Next, we introduce the four datasets used in this study and finally, we evaluate the performance degradation that occurs in OoD conditions in these four datasets.

### 3.1. Bias-controlled experiments

In a dataset there could be multiple biasing factors at the same time that can cause performance degradation. In the datasets in this study, we analyze either the orientation or illumination condition, as it allows to more clearly understand the effect of each individual factor. Thus, the datasets that we use contain several combinations of categories and conditions. We use 𝒞 to denote the set of all categories and 𝒩 the set of all orientation or illuminations conditions. Let $x^{\left(k\right)}$ be an image of the dataset and let $y^{\left(k\right)}≔\left(c^{\left(k\right)},n^{\left(k\right)}\right)$ be a tuple representing the groundtruth category (*i.e*., $c^{\left(k\right)}\in 𝒞$), and the orientation or illuminations condition (*i.e*., $n^{\left(k\right)}\in 𝒩$).

In order to evaluate the DNN’s OoD generalization capabilities, we train them in a dataset that follows a distribution that only contains a subset of all possible combinations, *i.e*., a subset of 𝒞×𝒩. Then, the DNN is evaluated with images from combinations that were not included in the training distribution. Let ℐ⊂𝒞×𝒩 be the set of combinations used to generate the InD combinations. We ensure that ℐ contains all categories and all conditions at least once (but not all combinations), such that we have images from all image categories and conditions in a balanced manner.

We use ${𝒟}^{\left(\text{InD}\right)}$ to denote the set of images that are InD, *i.e*., images whose label is in $ℐ,y^{\left(k\right)}\in ℐ$. Namely, the InD images dataset, ${𝒟}^{\left(\text{InD}\right)}$, is defined as in the following:

(1) $${𝒟}^{\left(\text{InD}\right)}≔\left\{\left(x,y\right)|y\in ℐ\right\}.$$

${𝒟}^{\left(\text{InD}\right)}$ is further divided into train dataset and validation dataset, which we denote as ${𝒟}_{\text{train}}^{\left(\text{InD}\right)}$ and ${𝒟}_{\text{val}}^{\left(\text{InD}\right)}$, respectively. The term InD accuracy refers to the DNN’s accuracy on ${𝒟}_{\text{val}}^{\left(\text{InD}\right)}$. The OoD dataset ${𝒟}^{\left(\text{OoD}\right)}$ is defined as

(2) $${𝒟}^{\left(\text{OoD}\right)}≔\left\{\left(x,y\right)|y\in \left(𝒞\times 𝒩\right)\setminus ℐ\right\}.$$

**Fig. 3.**
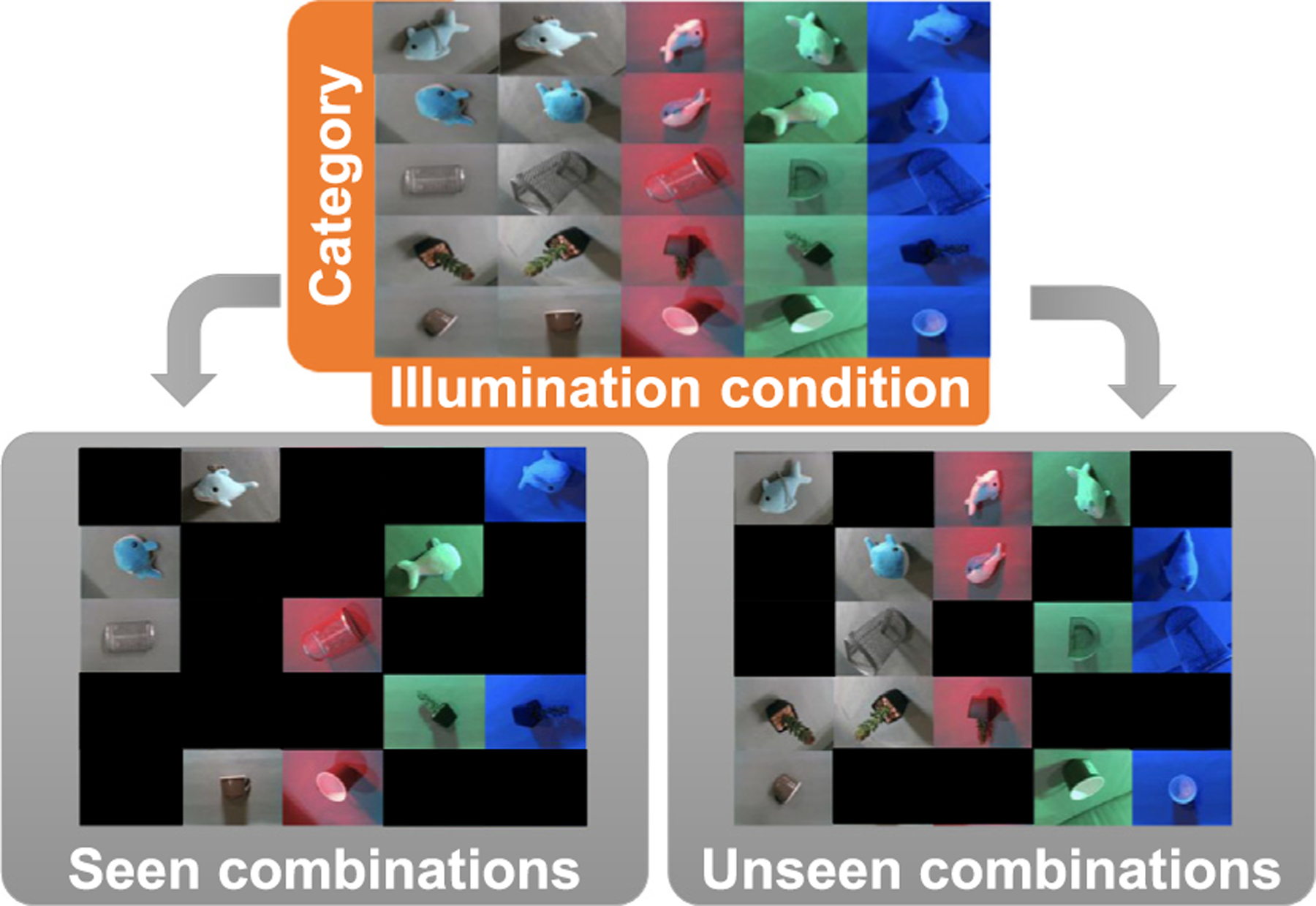
InD and OoD combinations for bias-controlled experiments. Each sample is a combination of a category and an orientation or illumination condition. We create a set of combinations called “InD combinations” and a set of combinations called “OoD combinations”. The ratio of InD combinations to all combinations is called InD data diversity. In addition, we create a train dataset $\left({𝒟}_{\text{train}}^{\left(\text{InD}\right)}\right)$ and an InD validation dataset $\left({𝒟}_{\text{val}}^{\left(\text{InD}\right)}\right)$ from samples included in the InD combinations, and an OoD test dataset $\left({𝒟}^{\left(\text{OoD}\right)}\right)$ from the samples included in the OoD combinations.

The term OoD accuracy refers to the accuracy on the OoD dataset ${𝒟}^{\left(\text{OoD}\right)}$. Fig. 3 also illustrates how to split all dataset to OoD dataset and InD dataset. Appendix A elaborates this bias-controlled experiments.

We also define the InD data diversity of a dataset as #(ℐ)/#(𝒞×𝒩), where #(⋅) denotes a number of elements. Thus, the data diversity measures the portion of combinations included in the training distribution. To directly compare the effect of the InD data diversity on the OoD accuracy, we vary the InD data diversity such that the combinations in the distributions of lower InD data diversity are included in the combinations of higher InD data diversity, while keeping the training set size constant, *i.e*., $\#\left({𝒟}_{\text{train}}^{\left(\text{InD}\right)}(ℐ)\right)$ is constant for all InD data diversity. These restrictions allows us to evaluate the performance of the DNN only by the difference in InD data diversity, not by the difference in the amount of combinations or training examples.

### 3.2. Datasets

There is a plethora of benchmarks for OoD generalization by now, *e.g*., Gulrajani and Lopez-Paz (2021), Hendrycks et al. (2021) and Koh et al. (2021). However, only few of these datasets are useful to investigate generalization to novel orientations and illuminations, as only a few provide labels for category, orientation and illumination, and cover a wide range of conditions. We use the following four datasets, two of them are introduced in this paper. See Appendix B for further details than the ones provided in the following.

#### MNIST-Positions.

It is based on the MNIST dataset (LeCun et al., 1998). We created a dataset of 42 × 42 pixels with nine numbers by resizing images to 14 × 14 and placing them in one of nine possible positions in a 3 × 3 empty grid. We call this dataset the *MNIST-Positions* dataset. In our experiments, the digits are considered to be the category set, and the positions where the digits are placed is considered as the orientation. We use nine digits and nine positions. Samples are shown in Fig. 2(a). We used 54k images for ${𝒟}_{\text{train}}^{\left(\text{InD}\right)}$, 8K images for ${𝒟}_{\text{val}}^{\left(\text{InD}\right)}$ and 8K images for ${𝒟}^{\left(\text{OoD}\right)}$. Low, medium, and high InD data diversity are set to be 2/9, 4/9, and 8/9, respectively.

#### iLab-Orientations.

iLab-2M is a dataset created from iLab-20M dataset (Borji et al., 2016). The dataset consists of images of 15 categories of physical toy vehicles photographed in various orientations, elevations, lighting conditions, camera focus settings and backgrounds. The image size is 256 × 256 pixels. From the original iLab-2M dataset, we chose six categories (bus, car, helicopter, monster truck, plane, and tank) and six orientations. We call it iLab-Orientations. Samples are shown in Fig. 2(b). We resized each image to 64 × 64 pixels. We used 18K images for ${𝒟}_{\text{train}}^{\left(\text{InD}\right)}$, 8k images for ${𝒟}_{\text{val}}^{\left(\text{InD}\right)}$ and 8k images for ${𝒟}^{\left(\text{OoD}\right)}$. Low, medium, and high InD data diversity are set to be 2/6, 3/6, and 5/6, respectively.

#### CarsCG-Orientations.

CarsCG-Orientations is a new dataset that consists of images of ten types of cars in various conditions rendered by Unreal Engine. It includes ten orientations, three elevations, ten body colors, five locations and three time frames (daytime, twilight, night). We synthesize images with 1920 × 1080 pixels and resize them as 224 × 224 pixels for our experiment. We chose ten types of cars as categories and ten orientations for each of them. Samples are shown in Fig. 2(c). More samples are provided in Appendix B. In the experiment, we used 3400 images for ${𝒟}_{\text{train}}^{\left(\text{InD}\right)}$, 450 images for ${𝒟}_{\text{val}}^{\left(\text{InD}\right)}$ and 800 images for ${𝒟}^{\left(\text{OoD}\right)}$. Low, medium, and high InD data diversity are set to be 2/10, 5/10, and 9/10, respectively.

#### MiscGoods-illuminations.

MiscGoods-Illuminations is a subset of DAISO-10, a novel dataset collected for this study. The dataset consists of ten physical miscellaneous goods photographed using a robotic arm with five controlled illumination conditions, two ways of object placement, twenty object orientations, and five camera angles. Each image is 640 × 480 pixels in size. We chose five categories (stuffed dolphin, stuffed whale, metal basket, imitation plant and cup) and five illumination conditions as shown in Fig. 2(d). More samples are displayed in Appendix B. We resize the images to 224 × 224 pixels for our experiments. We used 800 images for train ${𝒟}_{\text{train}}^{\left(\text{InD}\right)}$, 200 images for ${𝒟}_{\text{val}}^{\left(\text{InD}\right)}$ and 400 images for ${𝒟}^{\text{(OoD)}}$. Low, medium, and high InD data diversity are set to be 2/5, 3/5 and 4/5, respectively.

### 3.3. OoD accuracy results

We now demonstrate that these four datasets are extremely challenging for DNNs as these achieve low accuracy in OoD conditions. We examine the performance degradation in three InD data diversity: low, medium, and high. Recall that we evaluate InD accuracy in ${𝒟}_{\text{val}}^{\text{(InD)}}$ and the OoD accuracy in ${𝒟}^{\left(\text{OoD}\right)}$. We use ResNet-18 (He, Zhang, Ren, & Sun, 2016) trained with ${𝒟}_{\text{train}}^{\left(\text{InD}\right)}$. The experimental setup is introduced in Section 6.1.

**Fig. 4.**
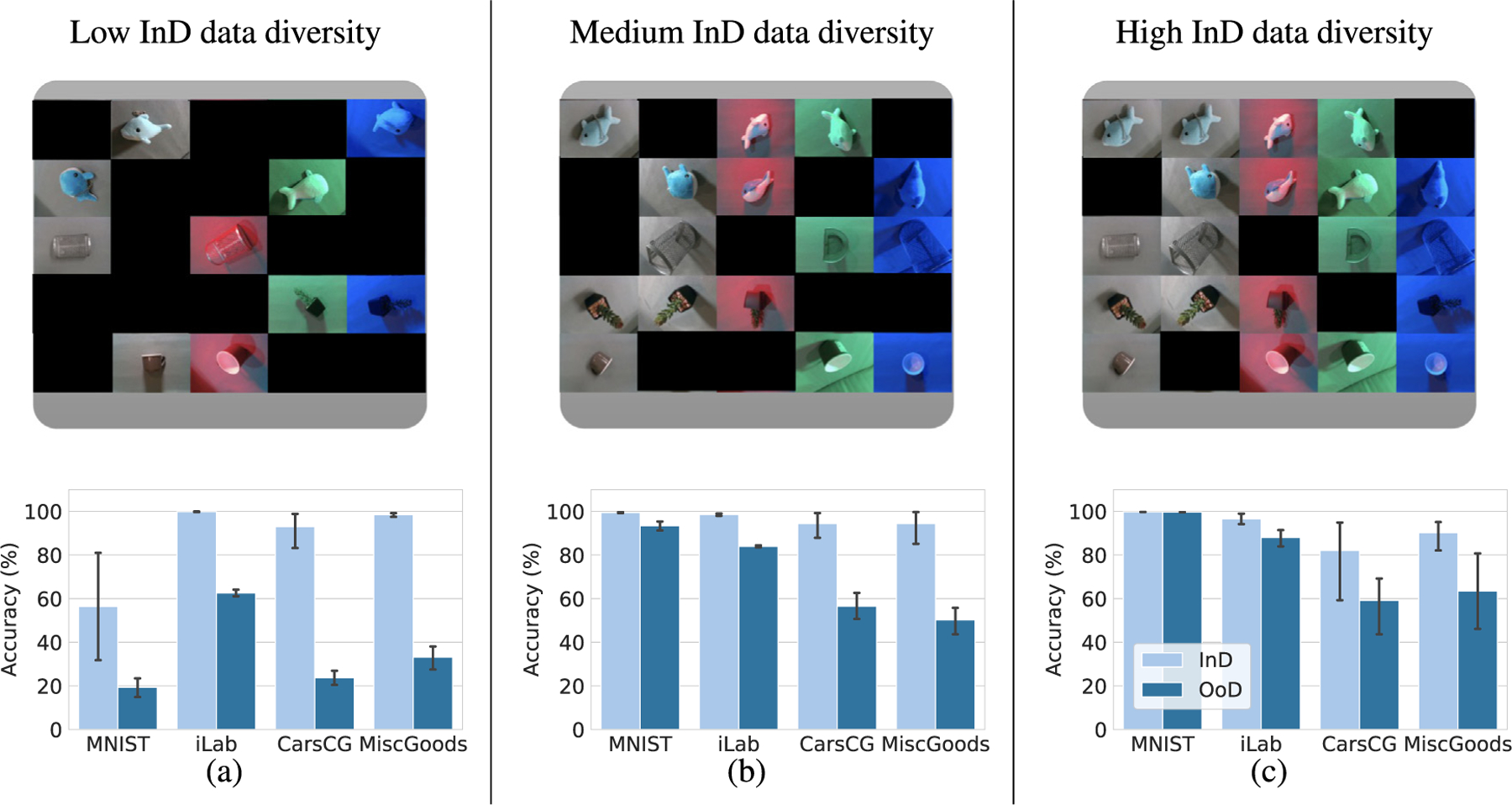
Performance degradation in the OoD conditions. Upper figures show the examples of InD combination in MiscGoods-Illuminations dataset to depict the InD data diversity of each experiment. Lower figures show InD or OoD accuracy of ResNet-18 in (a) low InD data diversity, (b) medium InD data diversity and (c) high InD data diversity performed on four datasets. Each experiment is conducted five times, and the mean and 95% confidence interval are reported. Sharp performance degradation in OoD accuracy is observed (*e.g*., between 40% to almost 80% is observed when the InD data diversity is low). These result shows the impact of a distribution shift from InD to OoD to the performance of a DNN.

Fig. 4 shows the OoD accuracy degradation regarding the four datasets ranging low to high InD data diversity. While the InD accuracy is more than 80% for all four datasets at almost all data diversities (except for MNIST-positions), the OoD accuracy showed a substantial degradation when the DNN was trained with low and medium InD data diversities. Between 20% to 70% performance degradation is observed in low InD data diversity in all four datasets. In medium InD data diversity, large performance degradation ranging from 10% to 50% is observed, and for high InD data diversity, there is more than 10% performance degradation in CarsCG-Orientations and MiscGoods-Illuminations datasets. Thus, dramatic drops of accuracy are observed in OoD conditions, which confirms that these benchmarks are very challenging for DNNs.

OoD accuracy is often overlooked in standard computer vision benchmarks and only InD is usually reported. This is usually due to the difficulty of measuring OoD accuracy. Our datasets enable evaluating OoD accuracy in a controlled way that facilitates understanding the different factors that may affect the OoD accuracy. The performance degradation in OoD conditions is expected when deploying application of deep learning. Recently, it has been reported that even a small amount of data bias can cause major performance degradation (Recht et al., 2018), and this is reconfirmed for our four datasets. Also, the drop of accuracy in our datasets is dramatic, specially for low InD data diversity. Our datasets allow to gain an understanding of the specific biasing factors in the dataset, *i.e*., orientation and illumination conditions, and analyze aspects such as the InD data diversity.

## 4. Three approaches to improve OoD accuracy

We now introduce the three approaches to address the performance drop of accuracy in OoD conditions, which are “late-stopping”, “tuning the batch normalization momentum” and “invariance loss”. These three approaches are independent on each other and tackle different aspects of the DNN training.

### 4.1. Late-stopping

The stopping criteria for training is known to have an impact on the DNNs performance (Caruana, Lawrence, & Giles, 2001; Cataltepe, Abu-Mostafa, & Magdon-Ismail, 1999; Yao et al., 2007b). In particular, stopping the training before convergence of the training accuracy, *i.e*., early stopping, is known to prevent overfitting in shallow classifiers (Prechelt, 1998). However, these results are with respect to InD accuracy, and little is known regarding the relation between the stopping criteria and OoD accuracy. We therefore run experiments with a large number of training epochs (up to 1000 epochs) in order to investigate any patterns. Fig. 1(a) shows the change of InD and OoD accuracy when ResNet-18 is trained with the medium InD data diversity. Surprisingly, the OoD accuracy, unlike the InD accuracy, continued to increase in performance after training during a large number of epochs. While recent work by Papyan et al. has shown that continuing training long after the classification error is zero, leads to important benefits such as improving robustness to adversarial attacks (Papyan, Han, & Donoho, 2020), we report for the first time that it also leads to improvements of OoD generalization. We denote the approach of continuing the training of a DNN after the convergence of InD validation accuracy as “late stopping”.

### 4.2. Tuning batch normalization

Batch normalization (BN) (Ioffe & Szegedy, 2015) is a method used to speed-up and stabilize the training of DNN networks through normalization of the layers’ inputs by re-centering and re-scaling them. Batch normalization has also been reported to act as a regularizer (Luo, Wang, Shao, & Peng, 2019). Yet, in OoD conditions the statistics of the dataset may change and hence, the statistics used to normalize the layer may not be valid anymore. Previous works have pointed out that in OoD conditions batch normalization needs to be adjusted (Schneider et al., 2020; Xie et al., 2020). Thus, it is reasonable that batch normalization also needs adjustment to help improving generalization to OoD orientations and illuminations, but this has not been studied so far.

Batch normalization uses the so called moving average to recenter the layer’s input. Let $v_{\text{ma}}(t)$ be the moving average at training step t. The moving average is updated at each training step in the following way:

(3) $$v_{\text{ma}}\left(t\right)=\left(\beta -1\right)v_{\text{mean}}\left(t\right)+\beta v_{\text{ma}}\left(t-1\right),$$

where $v_{\text{mean}}(t)$ is the mean activity over the batch of the tth training step, and β∈[0,1] is called momentum and balances the update of the moving average between $v_{\text{mean}}(t)$ and itself. Note that the only hyperparameter available for batch normalization is β, and we use this to adjust it. This value is often fixed, and we found that adjusting it is needed when the distribution of inputs changes. Usually, β is set to 0.9 or 0.99, which is the default value in standard deep learning frameworks. We use the default value 0.99 that is employed by the TensorFlow library (Abadi et al., 2016).

We investigated how the OoD generalization performance behaves depending on the value of the batch normalization momentum, β. Fig. 1(b) shows the learning curves of ResNet-18 trained on MiscGoods-illuminations with the medium InD data diversity. Experimentally, we found that the tuning momentum parameter, β, can have a significant positive impact on the OoD generalization performance. Generally, the default value of β=0.99 was too large for almost all cases in our experiments. We call this approach as tuning batch normalization or “tuning BN”.

### 4.3. Invariance loss

The “invariance loss” approach is intended to increase the invariance score that is introduced in Madan et al. (2022), which we explain in Section 5. This invariance score measures the degree of invariance in the neural activity of intermediate layers, and previous works have shown that DNNs that generalize better to OoD conditions have developed larger degrees of invariance in the intermediate layers.

Concretely, we encourage the emergence of invariant representations by taking pairs of images that belong to the same category and enforce that the neural activity is as similar as possible. To do so, we use the Euclidean distance between the activities of neurons in an intermediate layer caused by the pairs of images, and add this as an additional loss term to the classification loss. Fig. 1(c) shows the scheme of this approach. Let $g\left(\cdot ;\theta_{g}\right)$ be the neural activity of a DNN’s intermediate layer, where $\theta_{g}$ are the parameters of the DNN before the intermediate layer. Let $f\left(\cdot ;\theta_{f}\right)$ be the output of the DNN given as input the intermediate layer, $g\left(\cdot ;\theta_{g}\right)$, where $\theta_{f}$ are the DNN parameters from the intermediate layer to the output of the network. Thus, the neural activity of the intermediate layer for an image x is $g\left(x;\theta_{g}\right)$ and the output of the whole network is $f\left(g\left(x;\theta_{g}\right);\theta_{f}\right)$. Let x be a training image, and let $x^{\prime }$ be another image that belongs to the same category as x, and is sampled from the training data $D_{\text{train}}^{\left(\text{InD}\right)}$ according to some sampling strategy (in our experiments, we use random sampling with uniform distribution across the training images of the same category). Thus, the invariance loss is expressed as

(4) $${\left\|g\left(x;\theta_{g}\right)-g\left(x^{\prime };\theta_{g}\right)\right\|}_{2}.$$

This term is added to the categorical cross entropy loss weighted with a hyperparameter that we call λ, such that the invariance loss term acts as a regularization term.

Note that the invariance loss is equivalent to the contrastive loss (Hadsell, Chopra, & LeCun, 2006) for positive examples in the context of metric learning, but it has not been used so far to improve generalization to OoD orientations and illumination conditions.

## 5. Invariant neurons for OoD generalization

We now revisit the mechanism at the individual neuron level of intermediate layers that previous works have suggested that facilitates OoD generalization, *i.e*., individual neurons being invariant to OoD conditions. This mechanism has been shown to explain the improvement in OoD accuracy with increased InD data diversity (Madan et al., 2022; Zaidi et al., 2020).

Neurons are interpreted as features detectors. A neuron is selective to some features when the neuron’s output value is high only when those features are present in the image. Invariance of neurons that are selective can be helpful for OoD conditions. Note that a neuron can be simply invariant by always having as an output the same value, which is not helpful for generalization. We refer to invariant neurons to those neurons that have a high degree of selectivity to some image features and invariance to OoD conditions, *i.e*., selectivity is assumed as a precondition to invariance.

For a given intermediate layer of the DNN, let $\alpha_{cn}^{j}$ be the average activity for the jth neuron over all images with the cth category and the nth orientation or illumination condition. For neuron j, the activity is 0 – 1 normalized. Let $c^{*j}$ be the category that a neuron j is most active on average, *i.e*., $c^{*j}≔{\text{argmax}}_{c}\sum_{n}\alpha_{cn}^{j}$. This is called preferred category. The selectivity score $S^{j}$ is defined as

(5) $$S^{j}≔\frac{{\overset{ˆ}{\alpha }}^{j}-{\overset{‾}{\alpha }}^{j}}{{\overset{ˆ}{\alpha }}^{j}+{\overset{‾}{\alpha }}^{j}},$$

where, ${\overset{ˆ}{\alpha }}^{j}≔\frac{1}{\#(𝒩)}\sum_{n}\alpha_{c^{*j}n}^{j}$ and ${\overset{‾}{\alpha }}^{j}≔\frac{\sum_{c\neq c^{*j}}\sum_{N}\alpha_{cn}^{j}}{\#(𝒞)(\#(𝒞)-1)}$ denote the average activity for the preferred category and for the remaining categories, respectively. This selectivity score ranges from zero to one and takes its maximum value in the case that the neuron average activity, $\alpha_{cn}^{j}$, is 0 for all categories except for the preferred category, *i.e*., the neuron is only active for the preferred category. The invariance score $I^{j}$ is defined as

(6) $$I^{j}≔1-\left(\underset{n}{\text{max}}\;\alpha_{c^{*j_{n}}}^{j}-\underset{n}{\text{min}}\;\alpha_{c^{*j_{n}}}^{j}\right),$$

and it also ranges from zero to one and takes the maximum in the case that the average activity, $\alpha_{cn}^{j}$, takes the same value for the preferred category regardless of the orientation and illumination conditions.

Finally, we define the SI score of a neuron as the geometric mean of the selectivity and invariance scores, *i.e*., $\sqrt{S^{j}I^{j}}$. Neurons that have a larger SI score are active for specific categories independently on the orientation and illumination conditions. Networks with neurons that have larger SI scores have been observed to generalize better in OoD conditions. In order to provide a score that summarizes the SI score across all neurons in the layer, we use the upper 20 percentile of the scores among all neurons. This is because not all neurons are required to have larger SI to improve OoD generalization, and we just take into account a portion of neurons with the highest SI score. In the experiments, we use this summary of the SI score across neurons to assess whether the three approaches we introduce yield improved OoD accuracy through improving selectivity and invariance.

Finally, note that there are other ways to analyze the neural activity, such as the popular t-SNE visualization (van der Maaten & Hinton, 2008). Our neural activity analysis is unique with respect to previous visualization works in that it can quantitatively assess the neural activity and directly relate it to OoD accuracy.

## 6. Experiments and analysis

We first introduce the experimental setup, and then report the OoD accuracy facilitated by the three approaches explained in Section 4. Finally, we analyze whether this boost of OoD accuracy is driven by selective and invariance mechanism revisited in Section 5.

### 6.1. Experimental setting

We apply the three approaches to improve OoD accuracy to ResNet-18 (He et al., 2016) and evaluate its effectiveness in the aforementioned datasets (MNIST-Positions, iLab-Orientations, CarsCG-Orientations, and MiscGoods-Illuminations). Standard ResNet-18 is adopted as the network for all experiments and we trained it in the standard manner. Namely, all neurons employ the ReLU activation function g(z)=max{0,z} (Dahl, Sainath, & Hinton, 2013) and Glorot uniform initializer (Glorot & Bengio, 2010) is adopted for the network weights initialization for all experiments. Adam (Kingma & Ba, 2015) is employed as the optimization algorithm. The pixels of images are normalized within 0 to 1 as a preprocessing for all datasets.

We run five trials in all cases and report mean accuracy and its 95% confidence interval. In each trial, the InD combinations are chosen randomly as long as they satisfy the conditions explained in Section 3.1, and the OoD combinations are created accordingly. Each of the four approaches, including baseline, is subjected to a hyper-parameter search before performing the five trials. We select the hyper-parameters in a different trial from the ones used to report OoD accuracy. In this reserved trial, we select the hyper-parameters with the highest OoD accuracy by grid search. For all tested approaches, we selected a learning rate in {0.1, 0.01, 0.001, 0.0001, 0.00001}, and other hyper-parameters depending in the approach. In Appendix C, we show that the results are not much sensitive to the hyper-parameter choice. In the following we detail the experimental setting of the different approaches.

#### Late-stopping.

The epoch size is set to 1000 epochs for late stopping, and 100 epochs for the other approaches, including baseline. We confirmed that 100 epochs are sufficient for convergence in InD accuracy by the preliminary experiments. For late stopping, we run as many epochs as computing resources allow (about a week of training).

#### Tuning batch normalization.

For tuning batch normalization, we perform a grid-search for β={0.01,0.1,0.5,0.9,0.99} in addition to the learning rate. For the other approaches, we use 0.99 as a momentum parameter β for batch normalization layer, which is the default value in TensorFlow.

#### Invariance loss.

Invariance loss is applied to the last ReLU activation layer “activation_17” which has 512 neurons. We keep fixed the pairs of images in which invariance is enforced, and we randomize the pairs from time to time. We perform a grid search to determine how frequently we randomize the pairs of images (the choices are randomizing every {10, 20, 50, 100} epochs). The weight of the invariance loss term, λ, is also selected via a grid search among the following values: λ={1.0,0.1,0.01,0.001,0.0001}. For more details we refer the reader to Appendix D.

### 6.2. Improvement of OoD accuracy

**Fig. 5.**
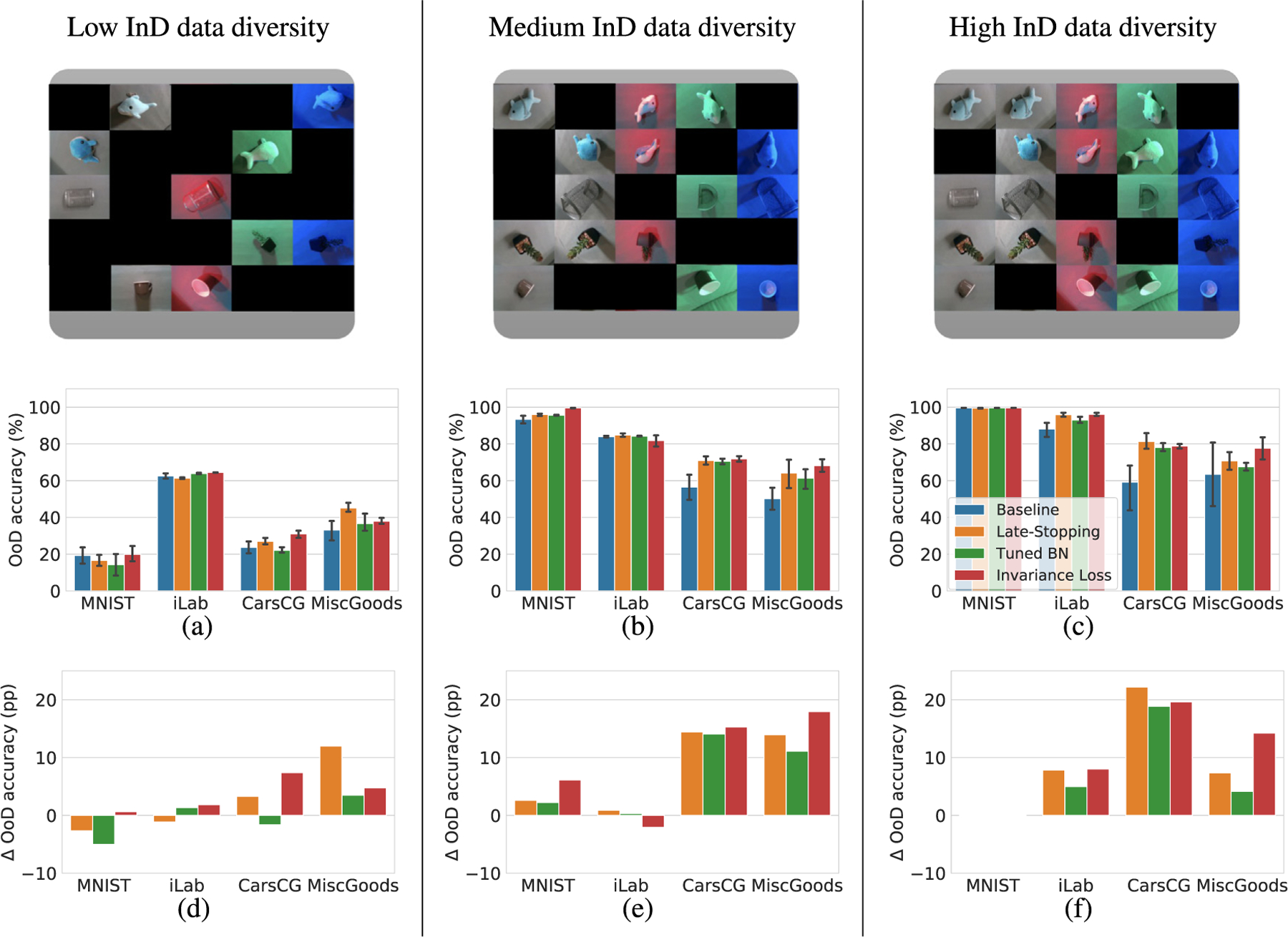
Performance improvement of the mean OoD accuracy. Top figures show the examples of InD combinations, *i.e*., InD data diversity, in MiscGoods-Illuminations dataset. (a), (b), and (c) show the mean OoD accuracy of the three approaches and the baseline for different InD data diversities. Error bars show 95% confidence interval. (d), (e), and (f) show the increase of the mean OoD accuracy by the three approaches over the baseline. The unit “pp” in figures denotes percentage points, *i.e*., the unit for the arithmetic difference of two percentages.

Fig. 5 compares the mean OoD accuracy between the baseline and the three approaches for all tested datasets and all tested InD data diversities. Looking at the case of the CarsCG-Orientations and MiscGoods-Illuminations datasets, we can see that the three approaches increase the mean OoD accuracy at almost all the data diversities. Comparing the three approaches, late stopping and invariance loss both achieves the best improvement rate in some combinations, and batch norm momentum does not achieve the best improvement in any combination. The highest improvement of 22.2% is achieved by late stopping with a high InD data diversity. The performance improvement across datasets and data diversities is remarkable. Only in iLab-Orientations dataset is relatively small, but for high InD data diversity in this dataset, all three approaches achieve better OoD accuracy than the baseline approach. For MNIST-Positions, all three approaches showed an improvement in performance with medium InD diversity. In Appendix E we report the learning curves and the InD accuracy for a more detailed depiction of the effects of the three approaches during training.

We also investigated whether the three approaches combined together are more effective than the best of three approaches applied alone. Thus, we trained networks using late-stopping, tuned BN and invariance loss together. We call this approach “three approaches together”. Another way of combining the three approaches is training networks with each approach alone and then selecting the best of the approaches using a validation set. We call this approach “best of three approaches alone”. The hyper-parameter tuning method of these combined approaches is detailed in Appendix F. Table 1 shows the comparison between these two combination approaches and also the baseline, *i.e*., the network trained without any approach to improve the OoD accuracy. The table reports the number times a method outperformed another method across all datasets and InD data diversity. The results show that using the best of the three approaches alone obtains the best results in the vast majority of experiments. Interestingly, the three approaches together performs worse than the baseline for more than half of the experiments. This indicates that the three approaches together interfere with each other and should not be used.

Finally, we compare the three approaches with state-of-the-art methods for OoD generalization, namely PGI (Ahmed et al., 2021) and CMMD (Li et al., 2018). Note that these methods were not introduced for OoD orientations and illuminations, but as a generic approach to OoD generalization. Recently, several researchers have pointed out that no single OoD algorithm can achieve high performance for all problem domains (Gulrajani & Lopez-Paz, 2021; Hendrycks et al., 2021; Wiles et al., 2022). In Appendix G we show that our three approaches outperform PGI and CMMD in OoD orientations and illuminations. We also show that PGI and CMMD can be combined with our approaches and lead to substantial improvements of PGI and CMMD’s accuracy. This result shows that our three approaches tackle complementary aspects from state-of-the-art methods for OoD generalization.

### 6.3. Analysis for selectivity and invariance mechanism

**Fig. 6.**
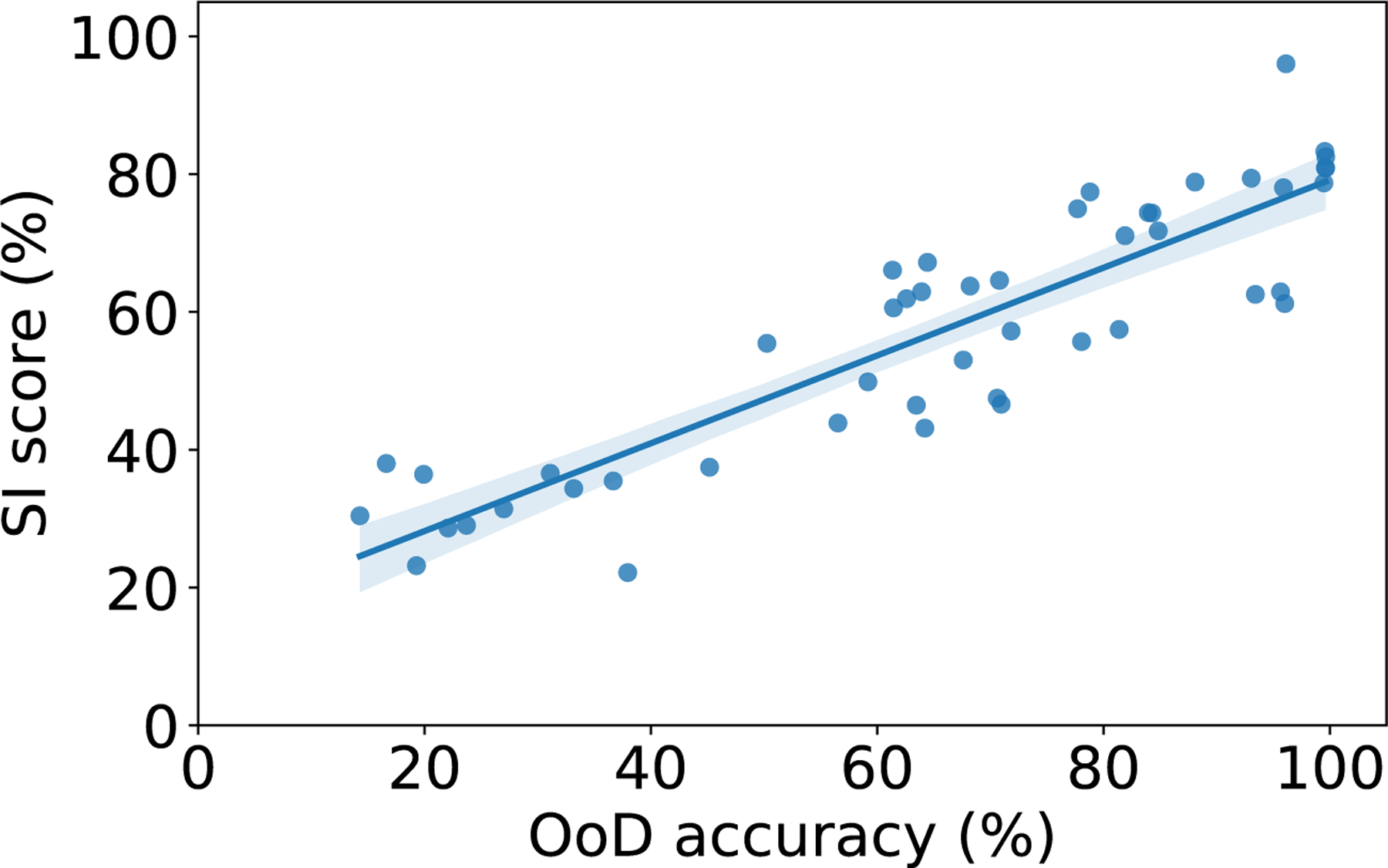
Correlation analysis. This figure shows the correlation between OoD test accuracy and SI score. The Pearson’s correlation coefficient is 0.891.

**Fig. E.9.**
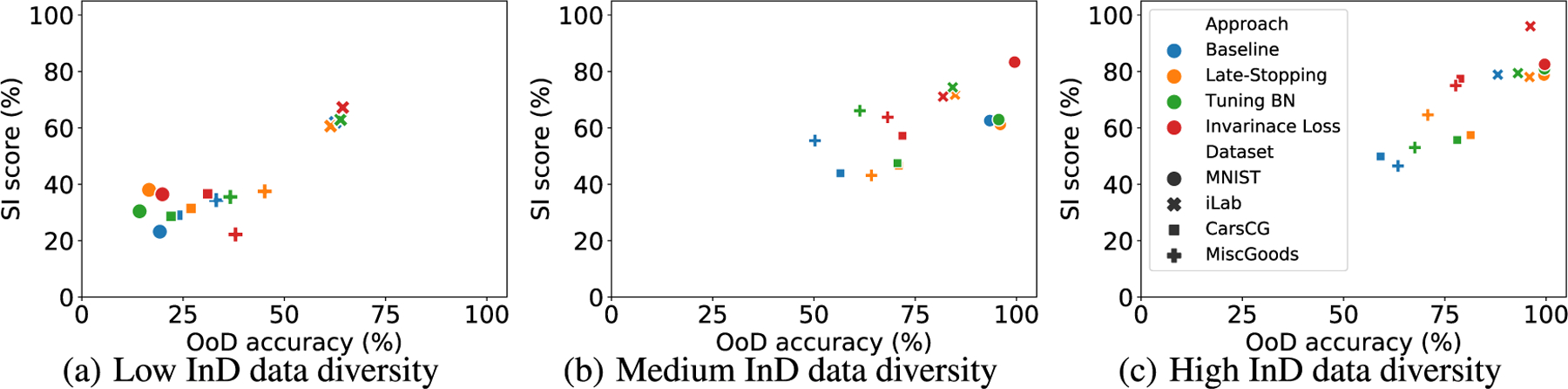
Overall results between IS scores and all combinations of (data diversity, dataset, approaches).

Fig. 6 shows the relationship between the SI score of the last ReLU layer and the OoD accuracy for all combinations of dataset, InD data diversity, and approach (details are provided in Fig. E.9). We can see that there is a large correlation between SI score and OoD accuracy (Pearson’s correlation coefficient is 0.891). While it has already been shown in Madan et al. (2022) that increasing the InD data diversity improves the OoD accuracy and the SI score, here we show for the first time that approaches that targets improving the OoD accuracy also yield increases of the SI score.

Next, we analyze the relationship between improvements of OoD accuracy and increases of the SI score. We investigate whether increases of the SI score always precede improvement of OoD accuracy, which serves to assess whether invariant representations drive OoD generalization in a more stringent way than the correlational analysis presented before. Let $P\left(\Delta_{\text{acc}}^{+}\right)$ be the probability that the OoD accuracy increases when using one of the three approaches to train the network, compared to not using it. Also, let $P\left(\Delta_{\text{SI}}^{+}\right)$ be the probability that the SI scores increases when using one of the three approaches, compared to not using it. The conditional probabilities between these two events provides insights regarding whether increases of the SI score precedes the improvements of the OoD accuracy. We calculate the probabilities by evaluating the frequency that the events happen across datasets and InD data diversities. We report them in Table 2.

We observe by analyzing $P\left(\Delta_{\text{acc}}^{+}\right)$ that the OoD accuracy increases very often with the three approaches, at least 75% of the cases. In particular, the OoD accuracy increased 91.7% of the cases for the invariance loss. The analysis of $P\left(\Delta_{\text{SI}}^{+}\right)$ shows that tuned BN and invariance loss increase the SI score 83.3% of the cases. This suggests that these two approaches tend to improve the SI score. For late-stopping this trend is not as strong. Yet, when analyzing $P\left(\Delta_{\text{acc}}^{+}|\Delta_{\text{SI}}^{+}\right)$, we observe that for the three approaches, increases of the SI score precede the improvements of OoD accuracy (this is in 83.3% (5/6), 80.0% (8/10) and 100% (10/10) of the cases for late-stopping, tuned BN and invariance loss, respectively). Note that the invariance loss directly encourages to increase the SI score, and when the SI score in fact increases, the OoD accuracy always has improved. Late stopping and tuning batch normalization momentum do not directly encourage to increase the SI score, but we observe that they do increase the SI score most of the cases, and when this happens, the OoD accuracy is also improved in more than 80.0% of the cases. Thus, these results suggest that the improvement of OoD accuracy is strongly driven by the increase of the SI score.

Finally, we observe by analyzing $P\left(\Delta_{\text{acc}}^{+}|\Delta_{\text{SI}}^{-}\right)$, that when the SI score has not increased after applying one of the three approaches, the OoD accuracy still improves in a non-negligible number of cases. This suggests the existence of another mechanism that can improve the OoD accuracy even if the selectivity and invariance mechanisms did not emerge. However, one possible limitation of this interpretation is that selectivity and invariance may have emerged but have not been captured by the SI score, because the SI score may not quantify the emergence of these mechanisms in the most precise way. Thus, we cannot make any assertion beyond the fact that it is unclear what are the neural mechanisms that facilitate OoD generalization when the three approaches do not manage to increase the SI score. This result motivates follow-up investigations.

In summary, in this study we provided evidence that the invariance and selectivity mechanism drives OoD generalization. Also, we found cases in which improvements of OoD generalization may not be preceded by the strengthening of the selectivity and invariance mechanism in the neural representations, which requires future work proposing novel mechanisms to explain these cases. We believe our experimental framework will facilitate such future discoveries.

Finally, in Appendix H we visualize the neural activity using t-SNE (van der Maaten & Hinton, 2008). This serves to illustrate the advantages of our analysis over this popular visualization tool. We observe that t-SNE displays neural invariance by having the samples of the same object categories close to each other. The trend is that for higher data diversity the samples are closer to each other, which is consistent with our analysis of neural invariance. Yet, our analysis of invariance provides more granular insights at the individual neuron level, rather than for an entire DNN layer as in t-SNE. Also, our analysis provides a quantitative assessment that directly relates with the OoD accuracy, unlike t-SNE, which only provides a qualitative assessment.

## 7. Conclusion

We have shown that late-stopping, tuning the batch normalization momentum parameter, and optimizing the invariance loss during learning lead to substantial improvements of the DNN recognition accuracy of objects in OoD orientations and illuminations (in some cases more than 20%). These improvements are consistent across four datasets, and different degrees of dataset bias. We also corroborated that the neural mechanisms of selectivity to a category and invariance to orientations and illuminations, at the individual neuron level, lead to the aforementioned improvements of OoD recognition accuracy. Namely, we found that in the majority of trials where any of the three approaches yield an increase of selectivity and invariance, resulted in improvements of the OoD recognition accuracy.

Nonetheless, our analysis also revealed that other mechanisms different from selectivity and invariance may also exists, as we observed that gains of OoD recognition accuracy were not preceded by an increase of the SI score in some trials. What are the neural mechanisms that drive OoD generalization in these cases remains as an open question for future work. Furthermore, there are also other novel questions derived from our results that motivate future works: Is there any effective way of combining the three approaches investigated in this paper that leads to even more improvements of OoD generalization? Are these approaches applicable to other factors beyond orientations and illumination conditions? How these approaches relate to biological learning systems? This paper is rather limited in providing answers to these fascinating questions that have cropped up ahead of us, and we hope that the substantial improvements of OoD recognition accuracy that we demonstrated in this paper motivate new research to address them.

Finally, we would like to highlight that poor OoD generalization is one of the issues of machine learning that needs to be urgently addressed in order to allow for safe and fair AI applications. We hope that this research serves as a basis for further improvements of OoD generalization.

## Figures and Tables

**Table 1:** Comparison of ways to combine the three approaches. We compare the best of the three approaches alone (*i.e*., training a network different times each with one of the three approaches alone, and then selecting the best of the three in a validation set), training with the three approaches together (*i.e*., training a network using the three approaches together), and the baseline (*i.e*., training the network without using any approach). Results compare how many times each of these strategies outperformed another strategy, across InD data diversities in each of the four datasets.

Comparison of OoD accuracy	MNIST	iLab	CarsCG	MiscGoods	Total
Best of 3 approaches alone vs. Baseline	3 vs. 0	2 vs. 1	3 vs. 0	3 vs. 0	11 vs. 1
3 approaches together vs. Baseline	1 vs. 2	1 vs. 2	1 vs. 2	2 vs. 1	5 vs. 7
Best of 3 approaches alone vs. 3 approaches together	3 vs. 0	2 vs. 1	3 vs. 0	1 vs. 2	9 vs. 3

**Table 2:** Analysis of the dependency between improvements of OoD accuracy and SI score. This table shows the relative frequency of improvement (+) or degradation (-) of the mean OoD accuracy $\Delta_{\text{acc}}$ or mean SI score $\Delta_{\text{SI}}$. Relative frequency P(x) is calculated by counting the number of cases that satisfy the condition $x\in \left\{\Delta_{\text{acc}}^{+},\Delta_{\text{SI}}^{+}\right\}$, and normalize it by total number of cases (*i.e*., 12, 3 possible InD data diversity × 4 datasets). Conditional relative frequency P(y∣x) is also calculated by counting the number combinations satisfying $y\in \left\{\Delta_{\text{acc}}^{+},\Delta_{\text{acc}}^{-}\right\}$ in the condition of $x\in \left\{\Delta_{\text{SI}}^{+},\Delta_{\text{SI}}^{-}\right\}$, and divide it by the number of combinations satisfying x. The first and second columns show the proportion of cases where the mean OoD accuracy $\Delta_{\text{acc}}^{+}$ and the mean SI score $\Delta_{\text{SI}}^{+}$ increased, respectively. The third column shows the proportion of cases where the mean OoD accuracy increased $\Delta_{\text{acc}}^{+}$ when the mean SI score increased $\Delta_{\text{SI}}^{+}$. The fourth column shows the proportion of cases where the mean OoD accuracy increased $\Delta_{\text{acc}}^{-}$ when the mean SI score increased $\Delta_{\text{SI}}^{-}$.

Approach	$P\left(\Delta_{\text{acc}}^{+}\right)$	$P\left(\Delta_{\text{SI}}^{+}\right)$	$P\left(\Delta_{\text{acc}}^{+}|\Delta_{\text{SI}}^{+}\right)$	$P\left(\Delta_{\text{acc}}^{+}|\Delta_{\text{SI}}^{-}\right)$
Late-stopping (%)	75.0 (9/12)	50.0 (6/12)	83.3 (5/6)	66.6 (4/6)
Tuned BN (%)	75.0 (9/12)	83.3 (10/12)	80.0 (8/10)	50.0 (1/2)
Invariance loss (%)	91.7 (11/12)	83.3 (10/12)	100.0 (10/10)	50.0 (1/2)
Total (%)	80.6 (29/36)	72.2 (26/36)	88.4 (23/26)	60.0 (6/10)

**Fig. B.1.**
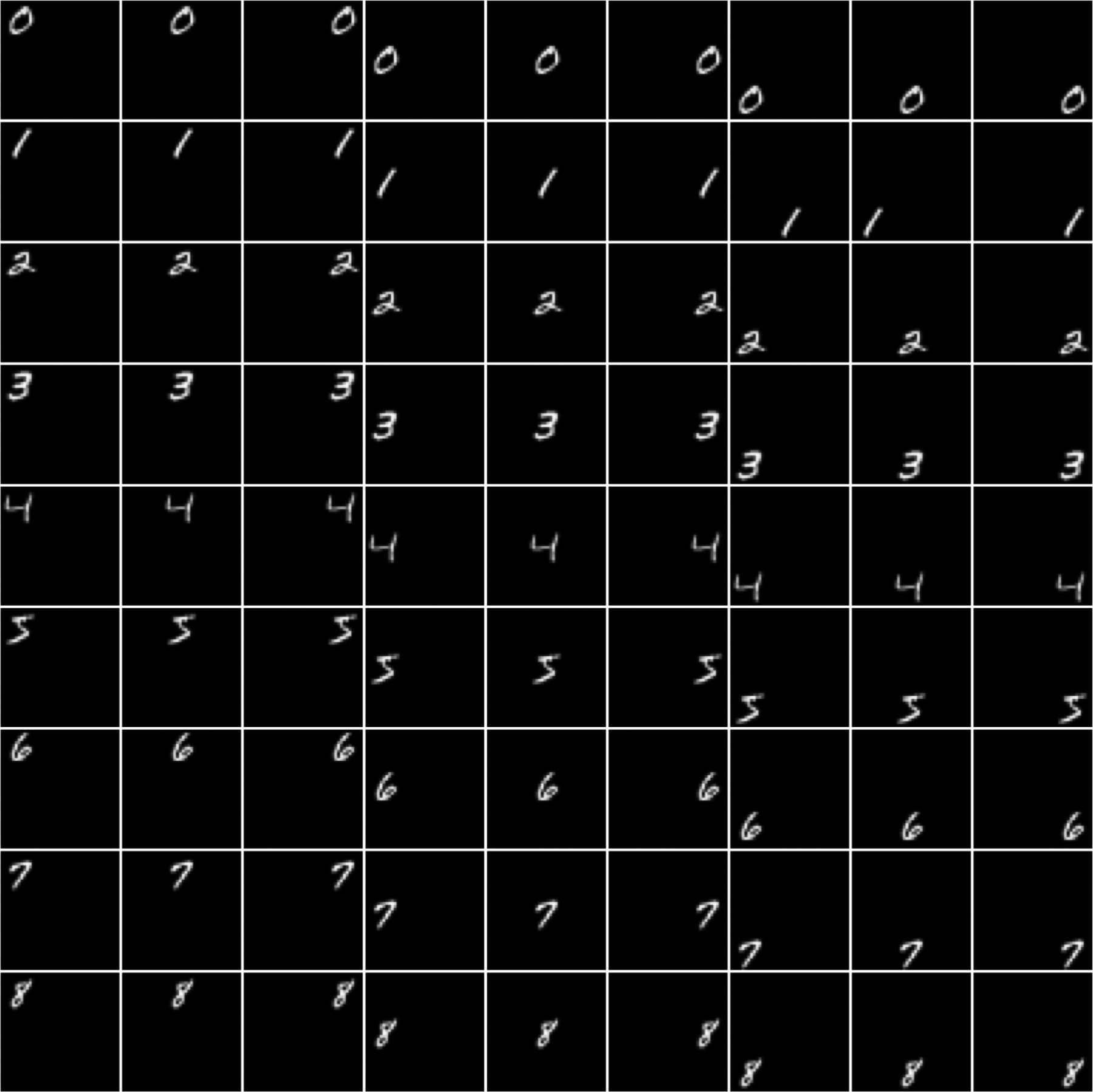
Sample images of MNIST-Positions dataset arranged in a grid pattern. Each row indicates the number as the object category. MNIST-Positions include nine numbers from 0 to 8. Each column indicates the positions as the condition category. There are 9 positions in this dataset.

**Fig. B.2.**
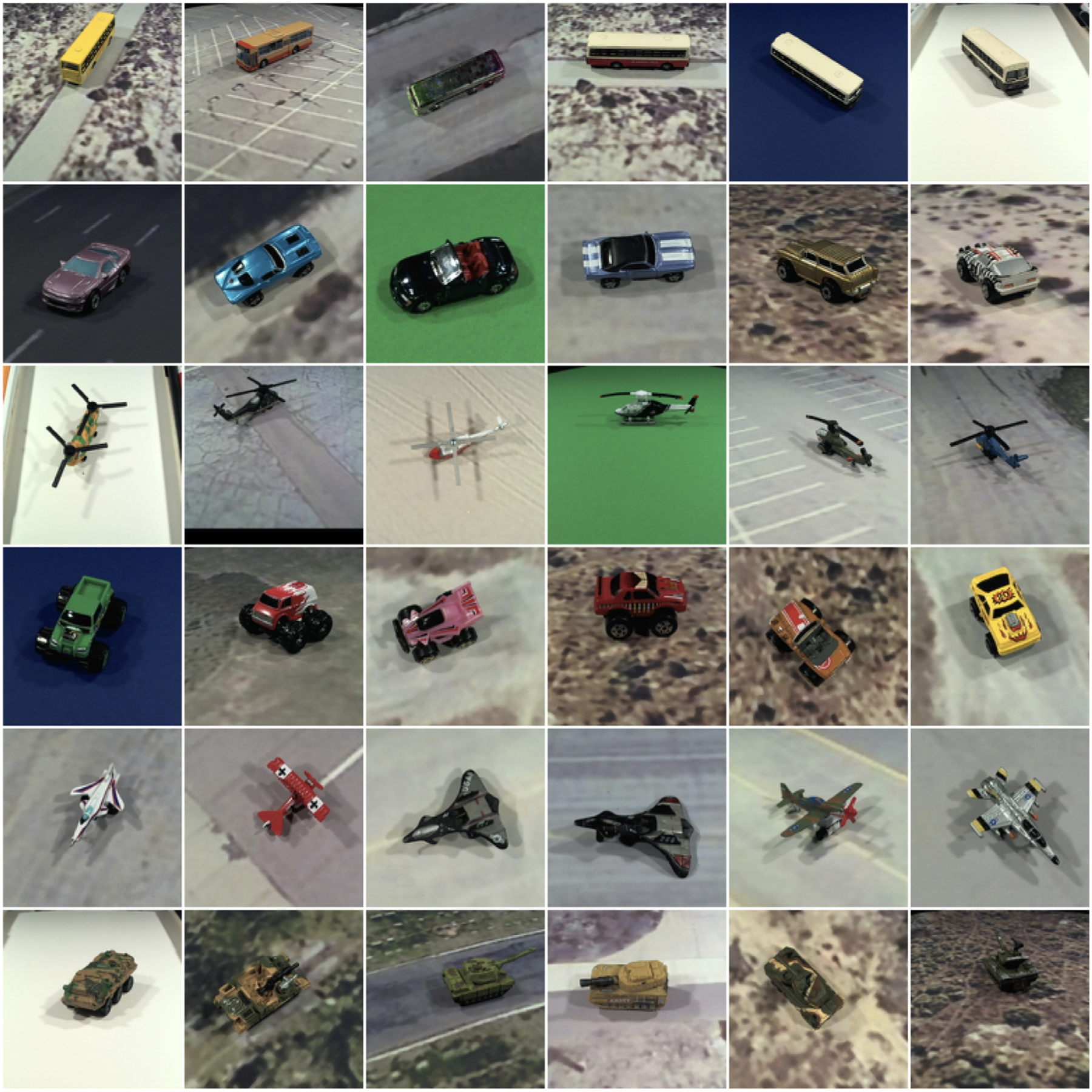
Sample images of iLab–Orientations dataset arranged in a grid pattern. Each row indicates the object category. iLab-Orientations include six object categories – bus, car, helicopter, monster truck, plane, and tank. Each column indicates the orientations as the condition category. There are 6 orientations in this dataset.

**Fig. B.3.**
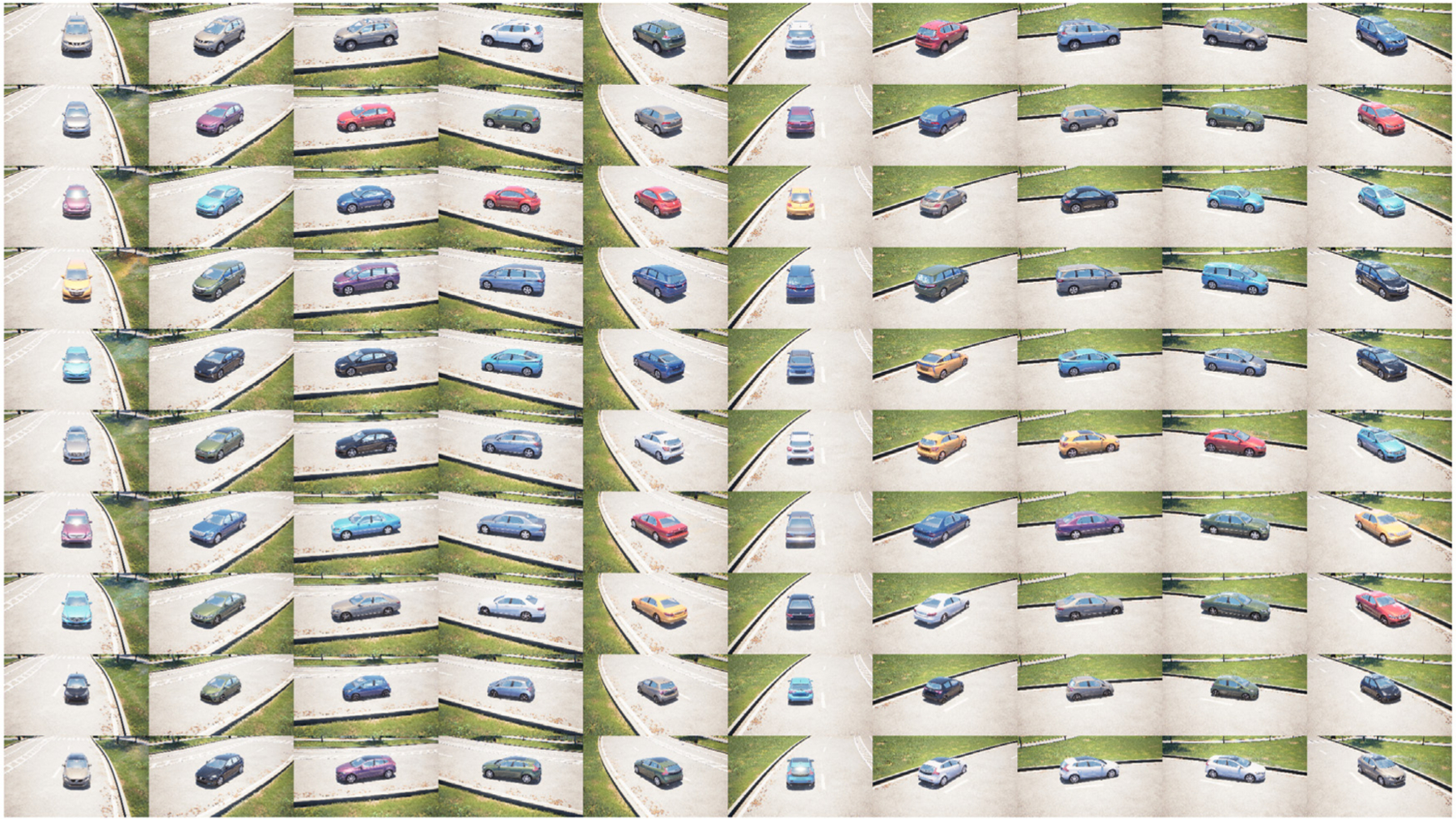
Sample images of each object category and orientation of CarCGs-Orientations. Each row indicates object categories – Nissan Rogue^®^, Volkswagen^®^ Golf, Volkswagen^®^ Beetle, Honda Odyssey^®^, Toyota Prius^®^, Mercedes Benz^®^ A-Class, Lexus^®^ LS, Mercedes Benz^®^ E-Class, Toyota Yaris^®^ and Volvo^®^ V40. Each column indicates the nuisance attribute categories, orientations from 0 to 324 degrees. These categories and orientations in this figure are used in our experiments.

**Fig. B.4.**
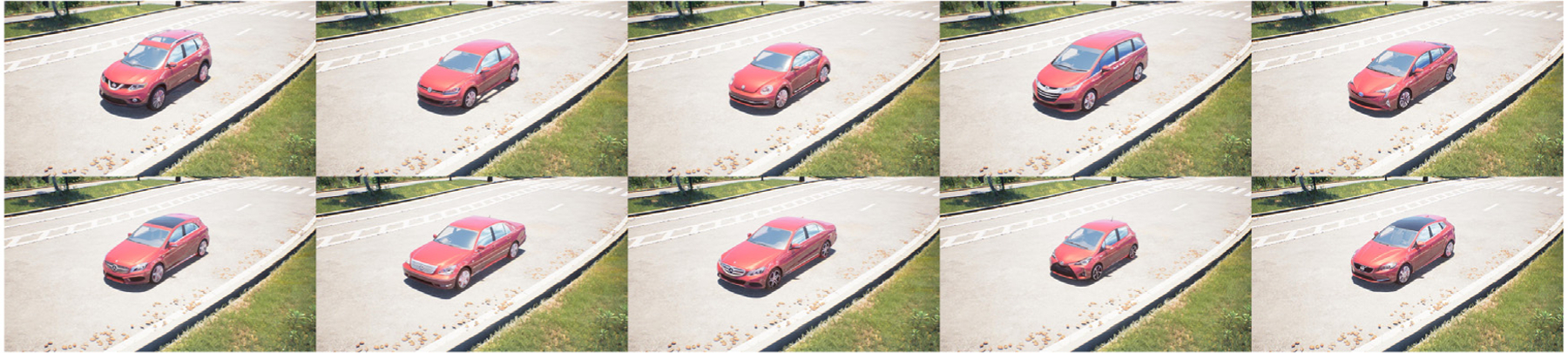
Sample images of ten object categories of CarsCG-Orientations – Nissan Rouge^®^, Volkswagen^®^ Golf, Volkswagen^®^ Beetle, Honda Odyssey^®^, Toyota Prius^®^, Mercedes Benz^®^ A-Class, Lexus^®^ LS, Mercedes Benz^®^ E-Class, Toyota Yaris^®^ and Volvo^®^ V40 are shown in this figure from the top left to the bottom right. All conditions except object category are fixed in this figure.

**Fig. B.5.**
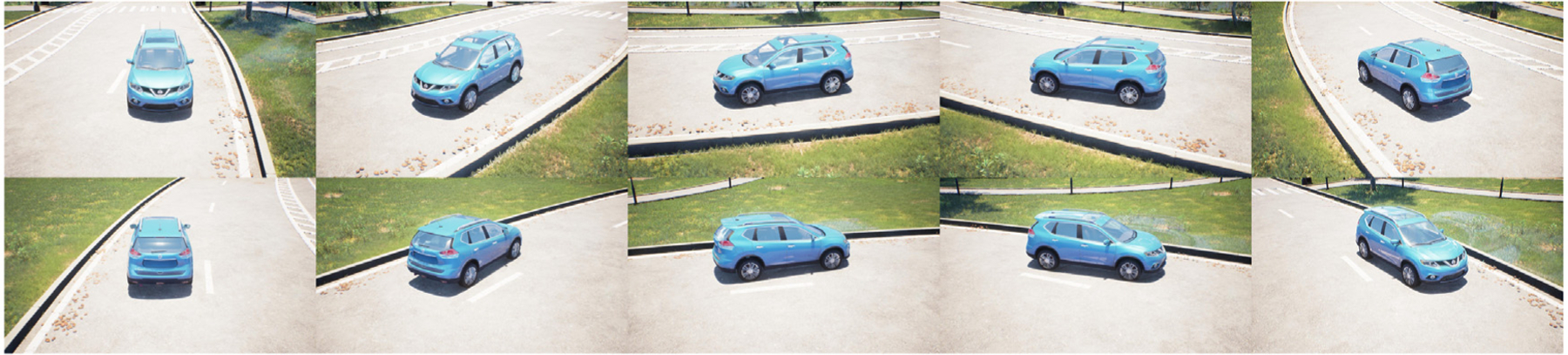
Sample images of ten orientations (condition categories) of CarsCG-Orientations. Ten orientations from 0 to 324 degrees are displayed from the top left to the bottom right. All conditions except orientation are fixed in this figure.

**Fig. B.6.**
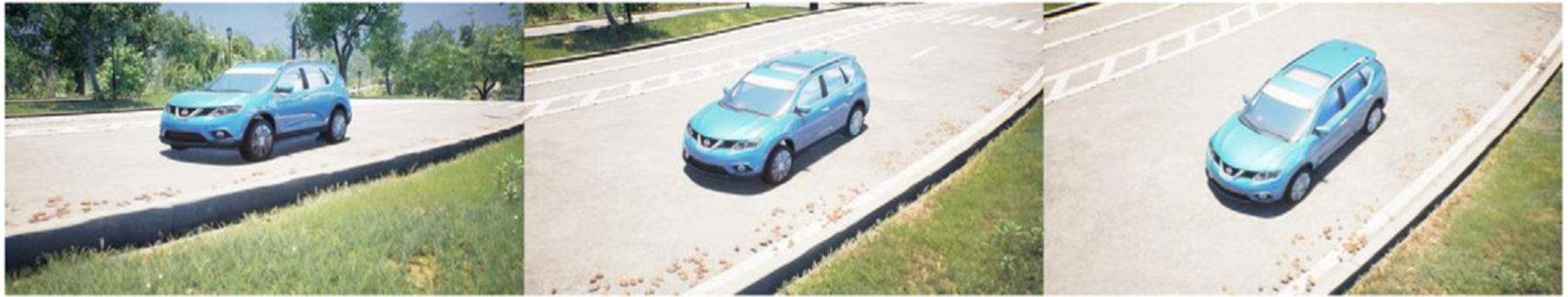
Sample images of the three elevation angles (10, 15, 30 degrees) of CarsCG-Orientations. Left figure is the image whose elevation angle is 10 degrees. Middle figure is the image whose elevation angle is 15 degrees. Right figure is the image whose elevation angle is 30 degrees.

**Fig. B.7.**
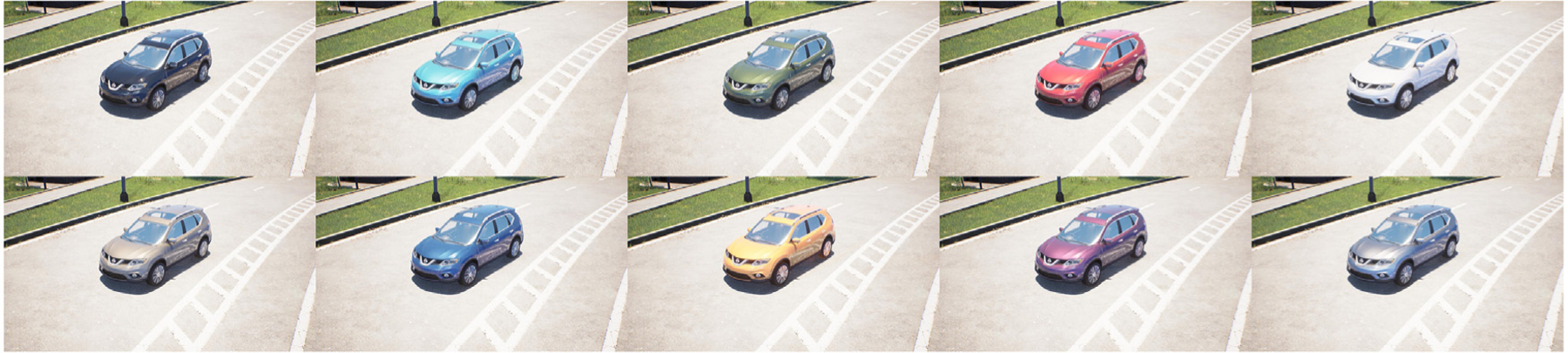
Sample images of cars painted in ten colors of CarsCG-Orientations. There are images painted in black, light blue, green, red, white, beige, dark blue, orange, plum, and silver from the top left to the bottom right.

**Fig. B.8.**
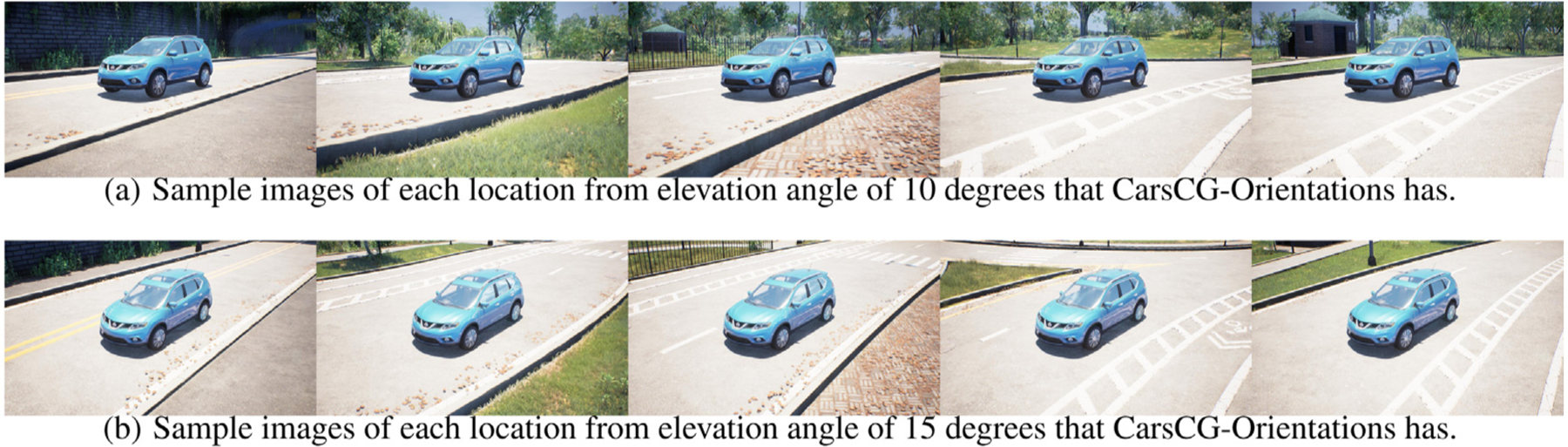
(a) is images of a car placed in five different locations taken from elevation angle of 10 degrees. (b) is images taken from elevation angle of 15 degrees. There are differences in texture of road and background.

**Fig. B.9.**
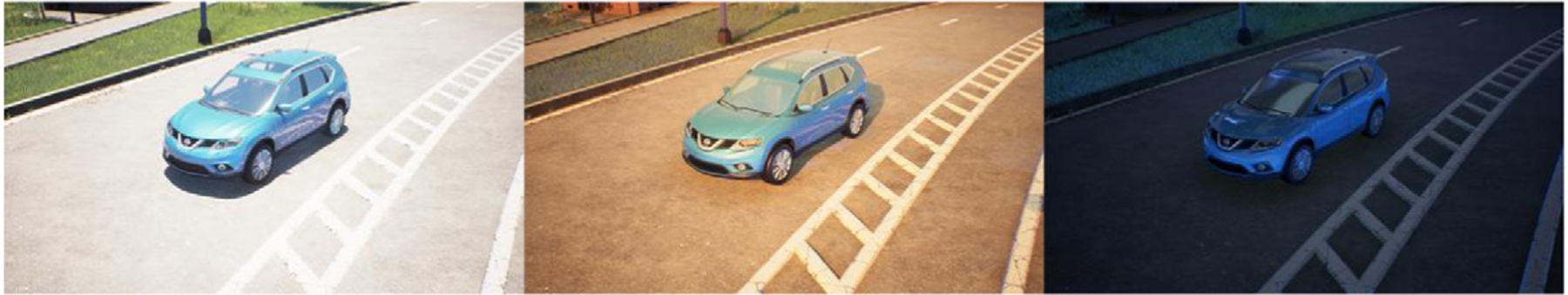
Sample images of each time slot of CarsCG-Orientations. Left figure is an image of car taken in the daytime. Middle figure shows an image of a car taken in the twilight. The color of the car is different from that in the left and right ones. It is caused by the twilight sunlight condition. Right figure shows an image of car taken at midnight. The color of the car is also different from left and middle ones.

**Fig. B.10.**
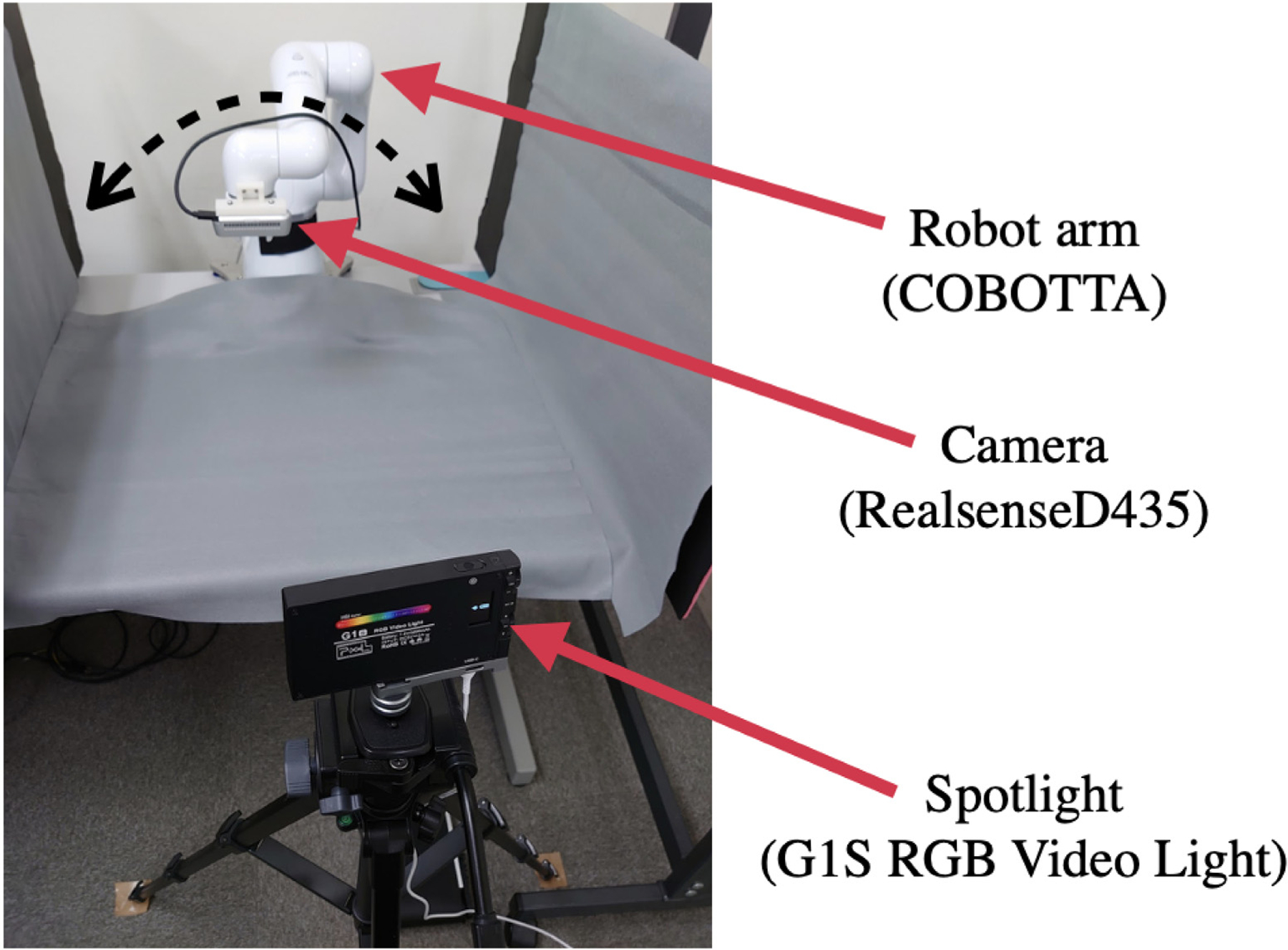
Robotic image capture system for MiscGoods-Illuminations. Dashed bidirectional arrow indicates the robot motion.

**Fig. B.11.**
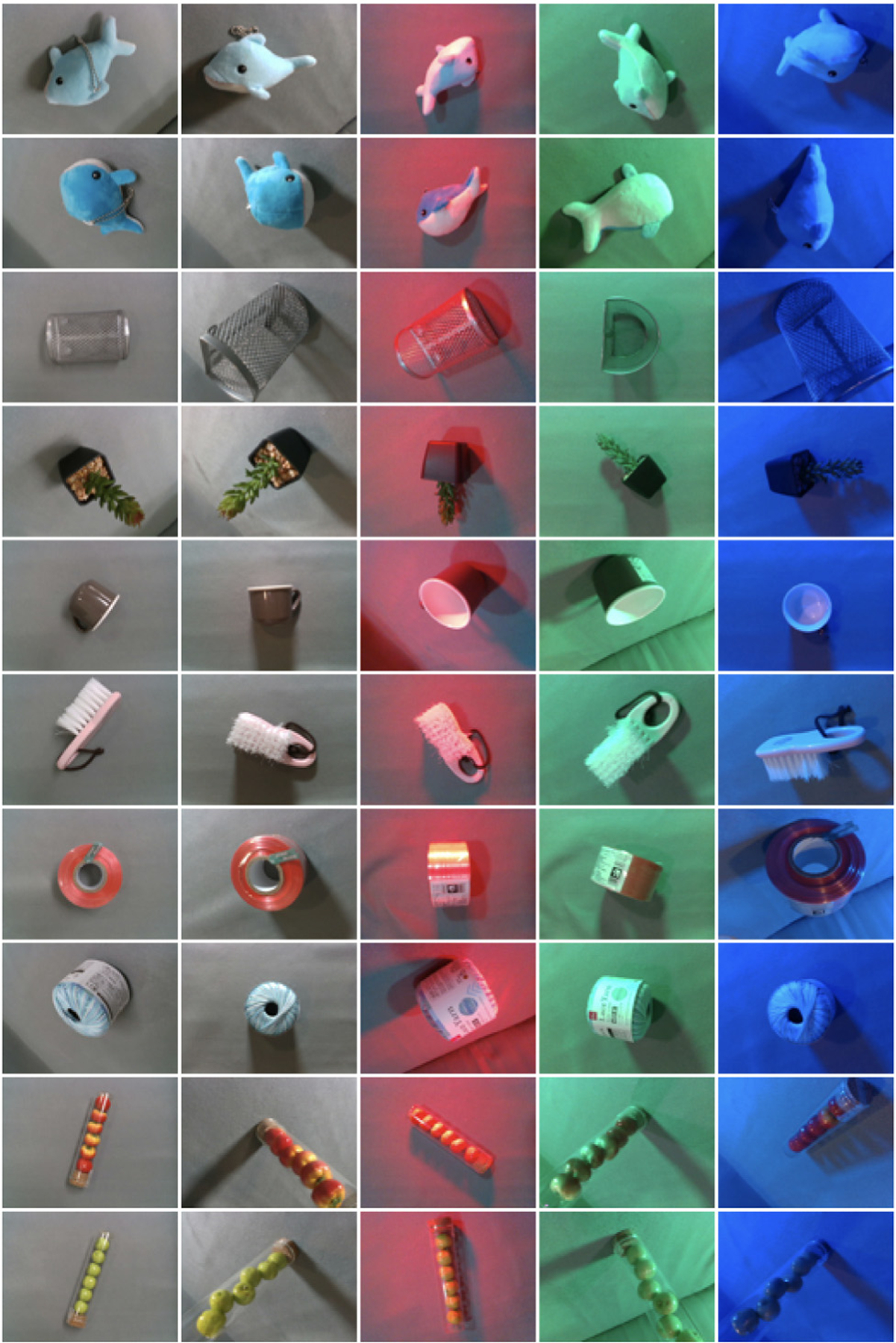
Sample images of each object category and illumination condition of MiscGoods-Illuminations are shown in this figure. Each row indicates object categories – stuffed dolphin, stuffed whale, metal basket, imitation plant, cup, cleaning brush, winding tape, lace yarn, bottled imitation tomatoes, and bottled imitation green apples. Each column indicates the condition categories, illumination conditions – ceiling light, white spotlight, red spotlight, green spotlight and blue spotlight. These five categories from the top and five illumination conditions are used as object categories and condition categories in our experiments.

**Fig. B.12.**
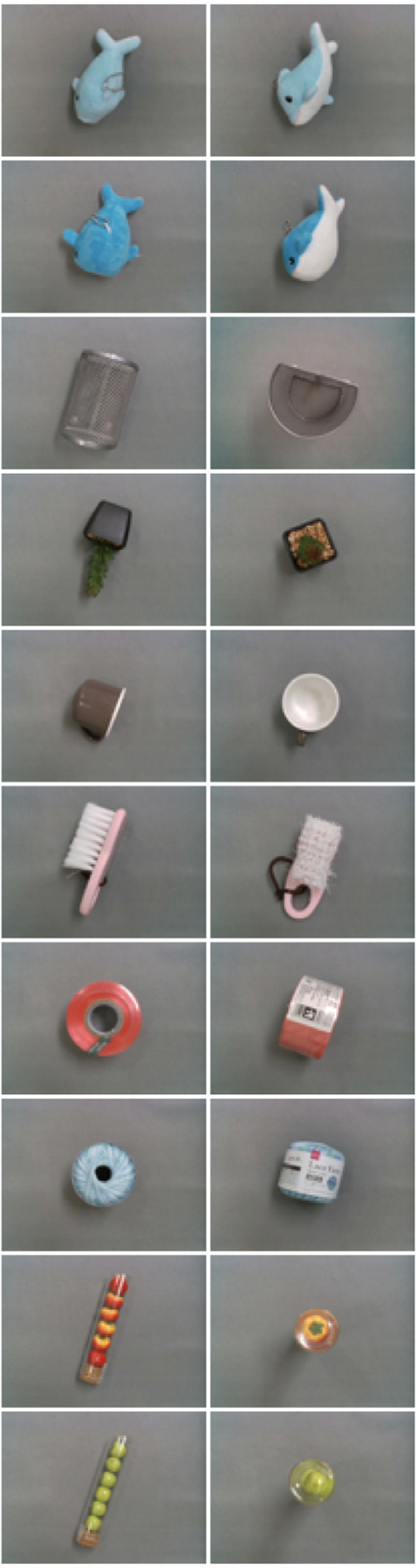
Sample images of MiscGoods-Illuminations with two ways of object placement. Each object has these two aspects as condition. The shapes of these objects in an image are changed by the way of object placement.

**Fig. B.13.**
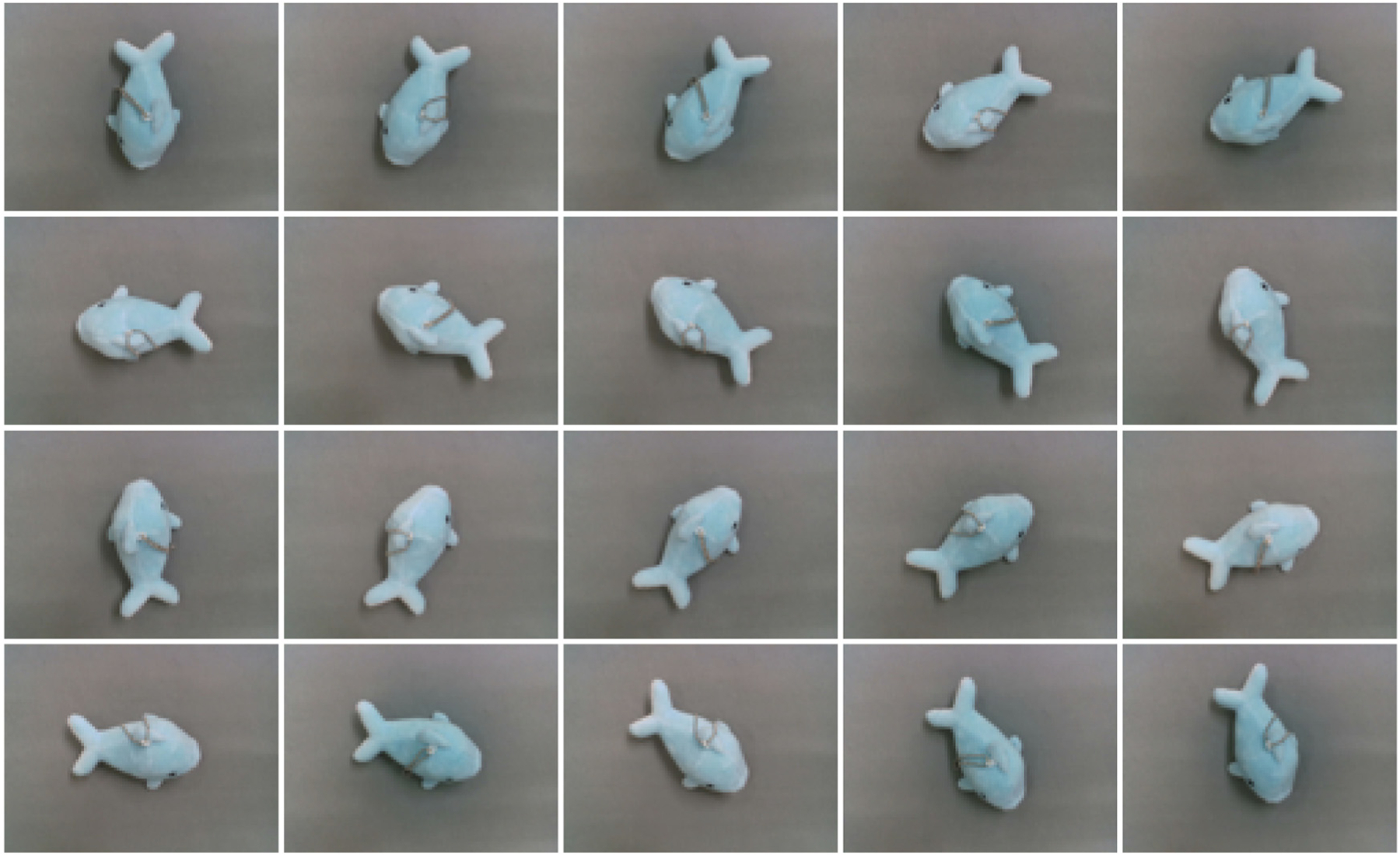
Sample images of each orientation of MiscGoods-Illuminations. 20 orientations from 0 to 342 degrees that the dataset has are shown in this figure from the top left to the bottom right.

**Fig. B.14.**
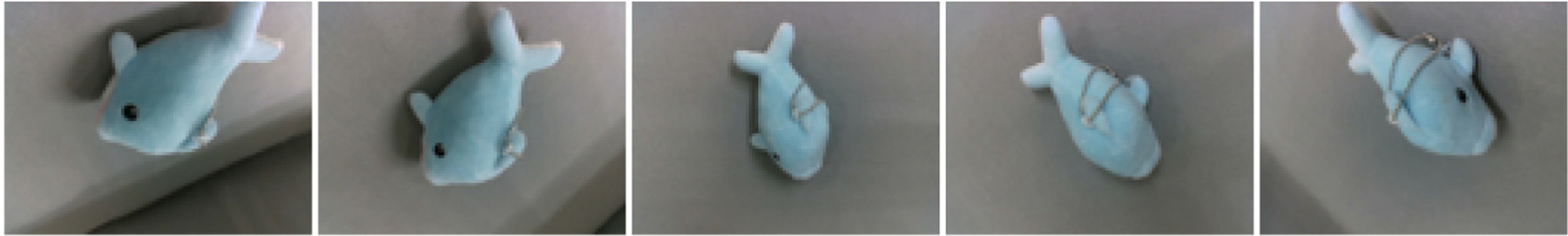
Sample images from each camera angles of MiscGoods-Illuminations. There are five angles in the dataset. The postures were defined so that the acquired image shows the entire object pose. These five camera angles are related to postures of robot arm that the camera is connected.

**Fig. D.1.**
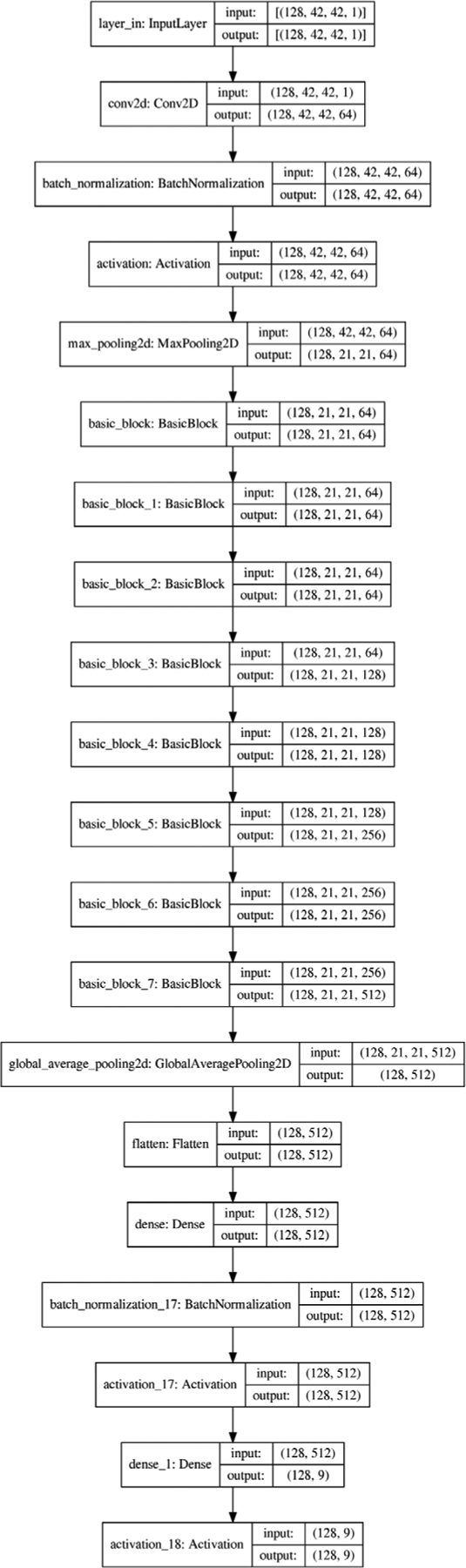
This diagram shows the whole architecture of our implementation of ResNet-18. The numbers in this diagram represent batchsize, height of image, width of image and channels. Therefore they change depending on the dataset. Current numbers correspond to MNIST-Positions. For instance, the numbers on the top of the diagram means (*batchsize, height, width, channels*) = (128, 42, 42, 1). Conv2D, Dense and BasicBlock mean a convolutional layer, a fully connected layer and a basic building block of ResNet, respectively.

**Fig. D.2.**
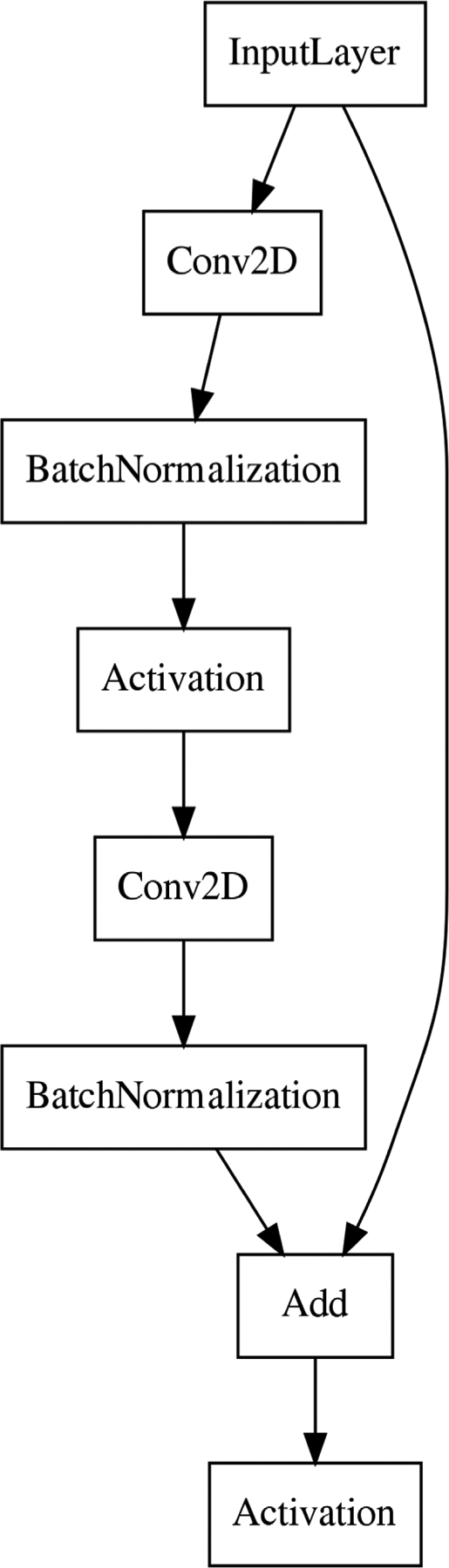
This diagram shows the architecture of BasicBlock in ResNet-18. Conv2D and Add mean a convolutional layer and a layer that simply add the two input values.

**Fig. E.1.**
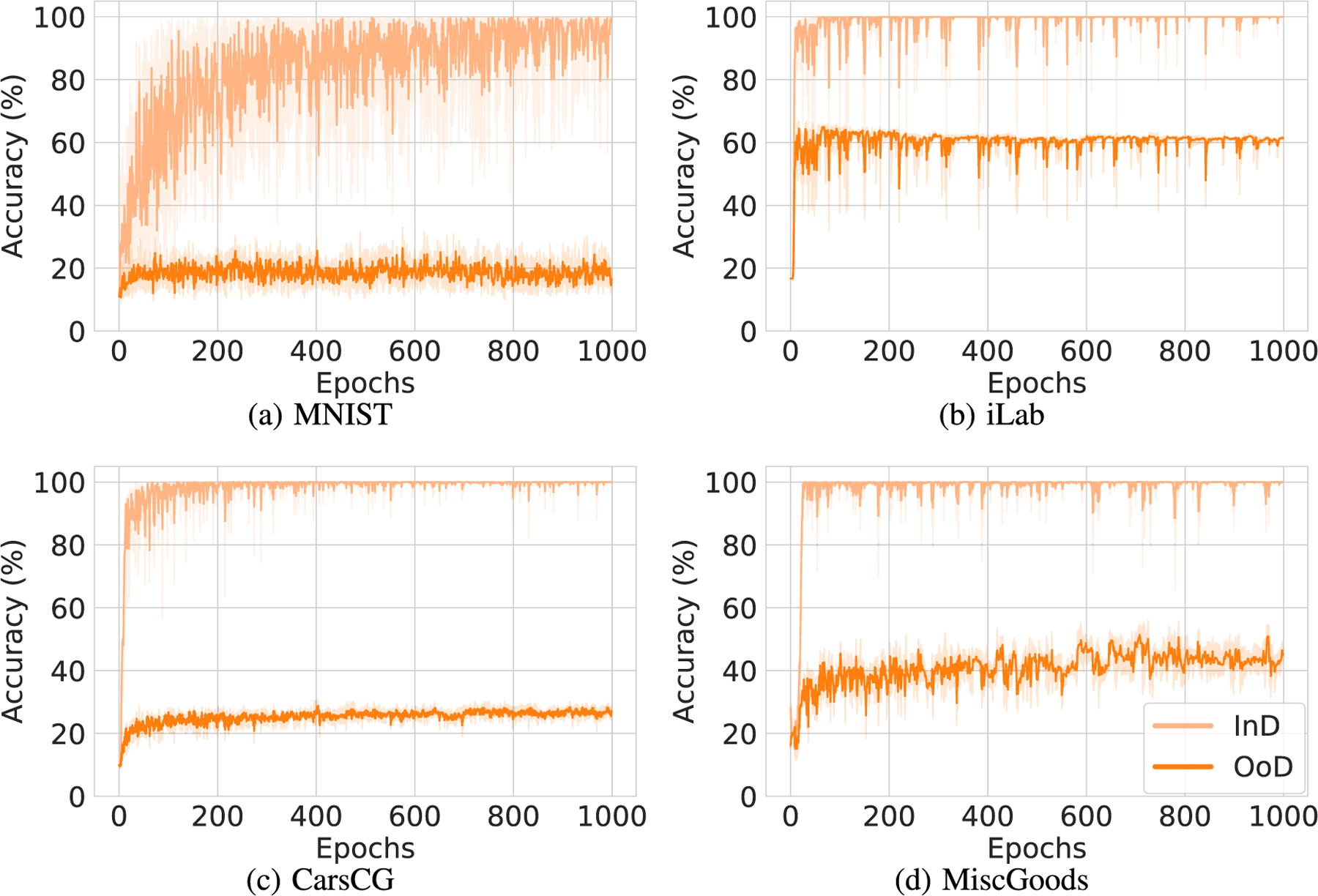
Late-stopping with low InD data diversity.

**Fig. E.2.**
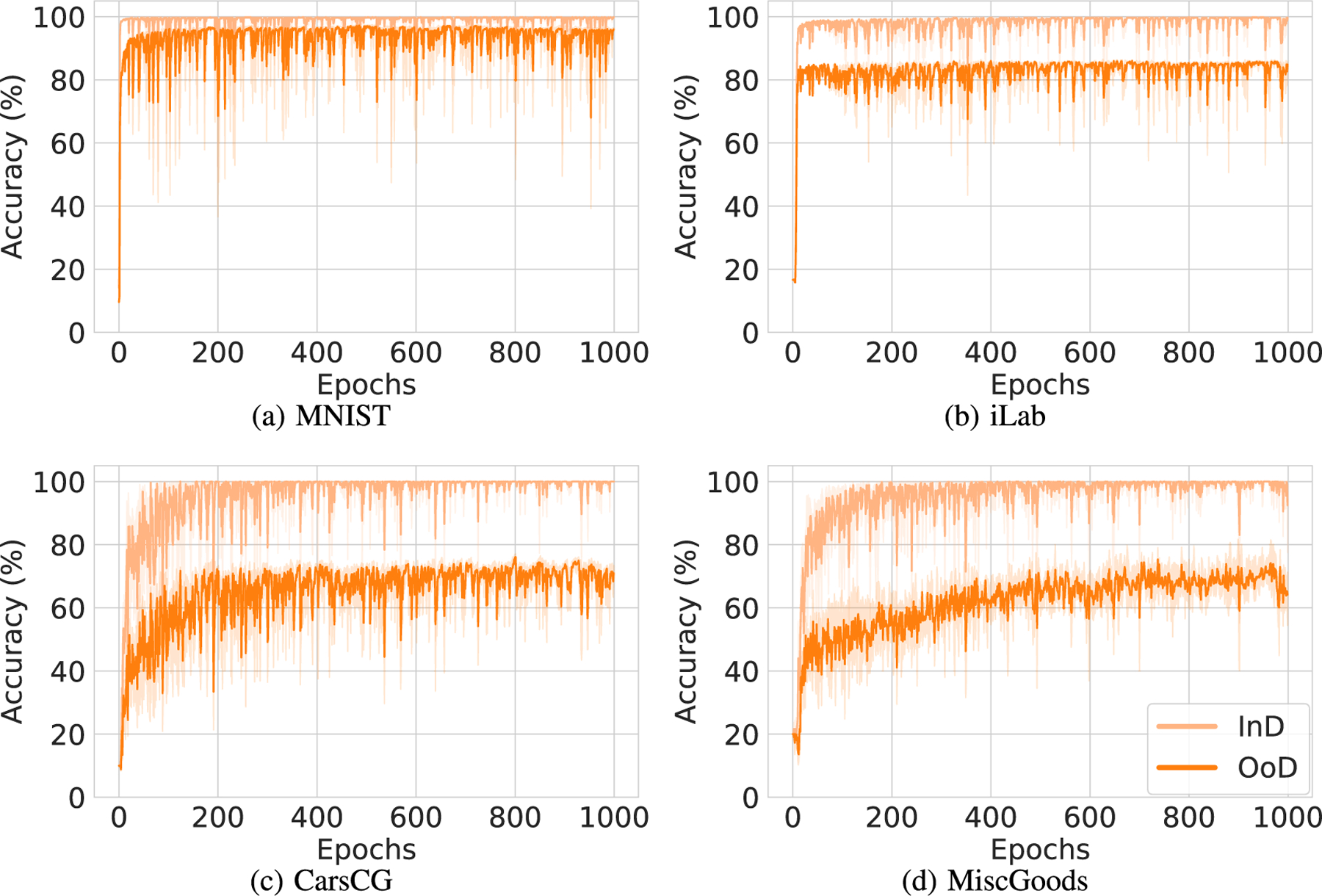
Late-stopping with medium InD data diversity.

**Fig. E.3.**
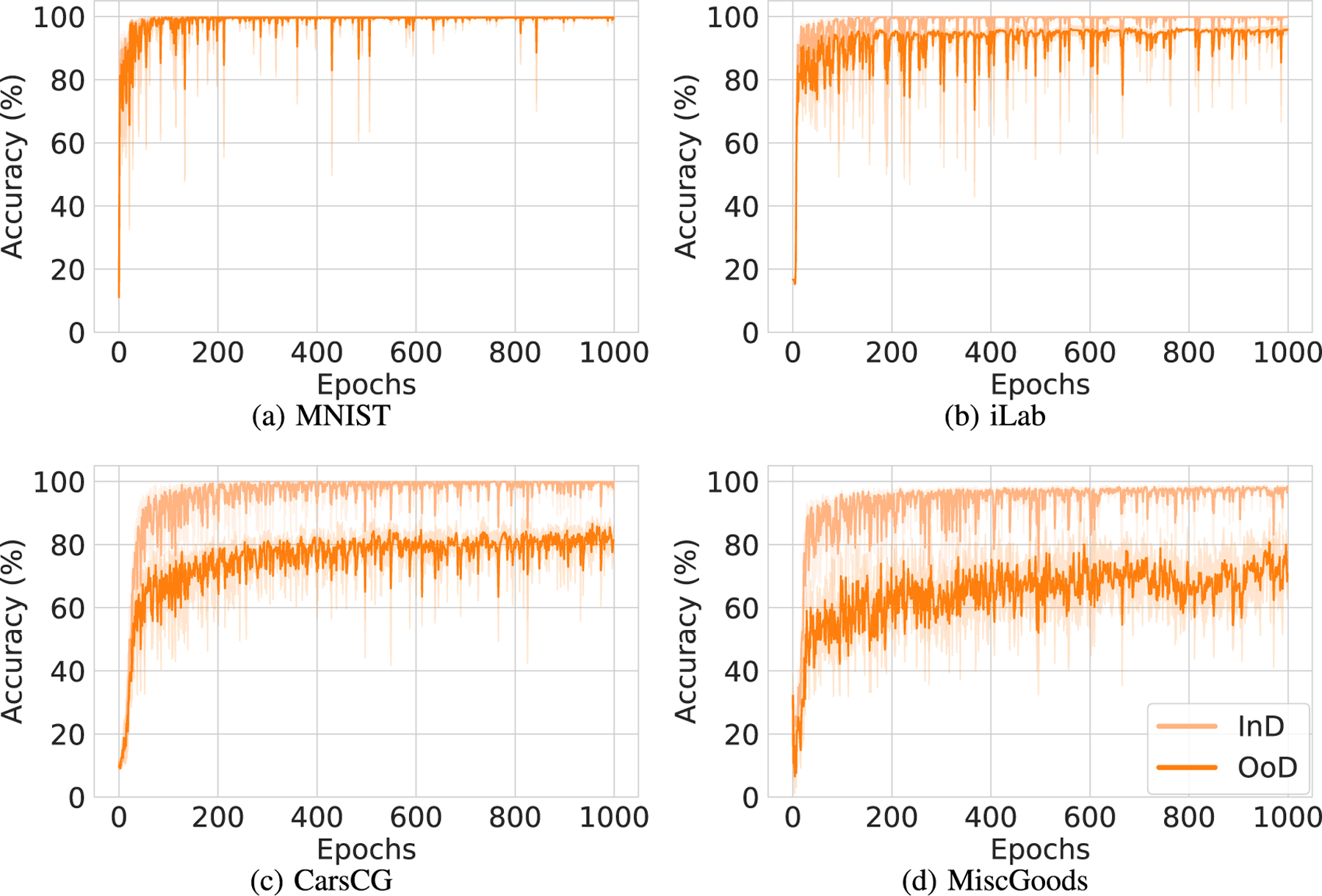
Late-stopping with high InD data diversity.

**Fig. E.4.**
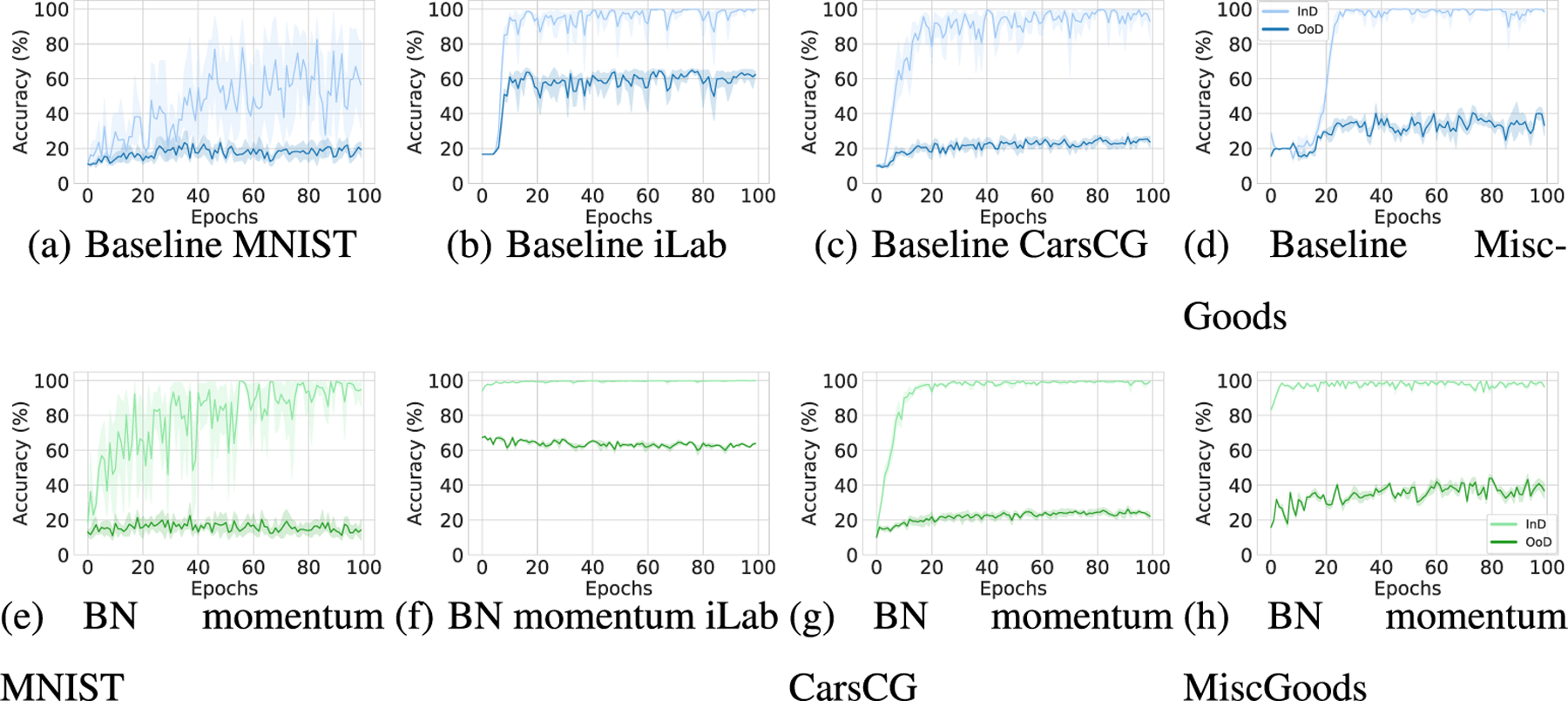
Baseline and BN momentum with low InD data diversity.

**Fig. E.5.**
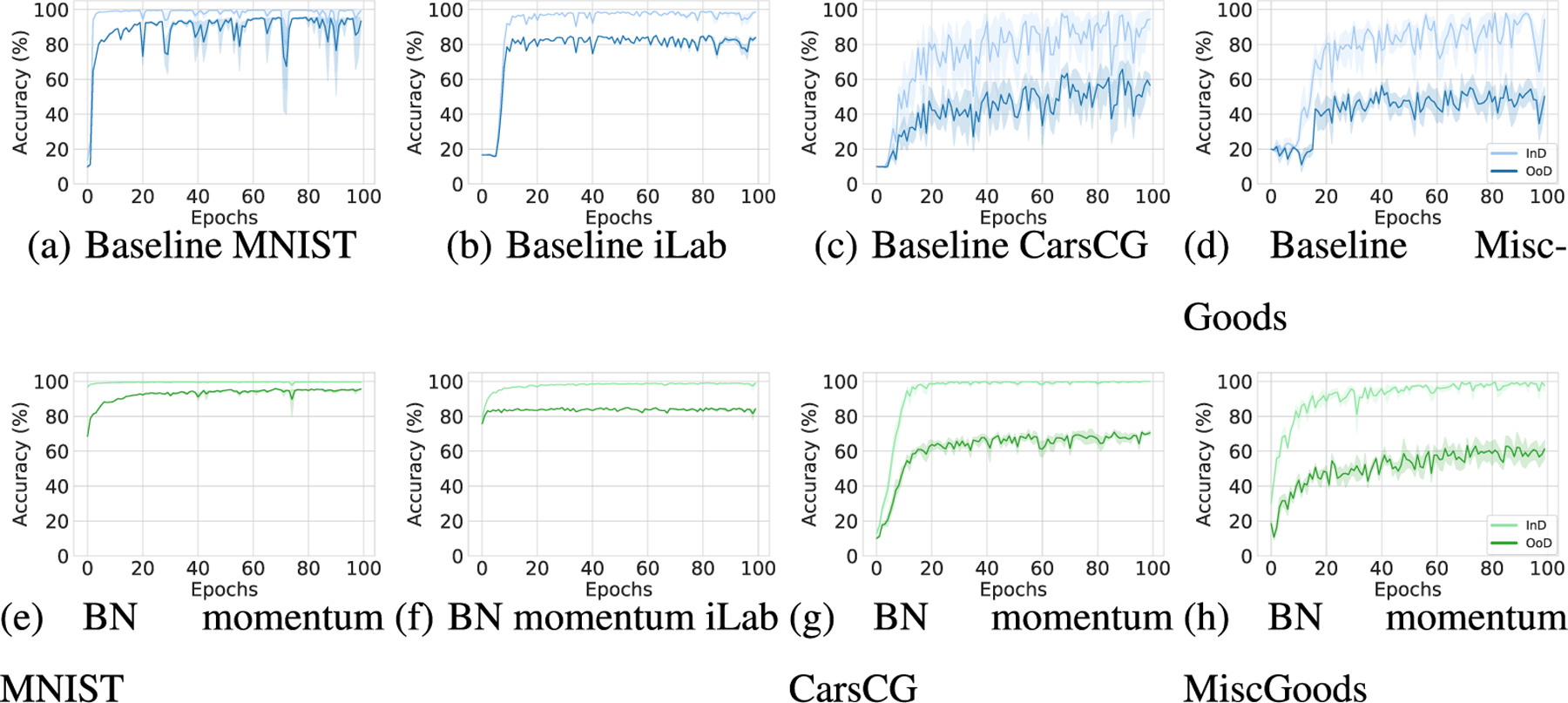
Baseline and BN momentum with medium InD data diversity.

**Fig. E.6.**
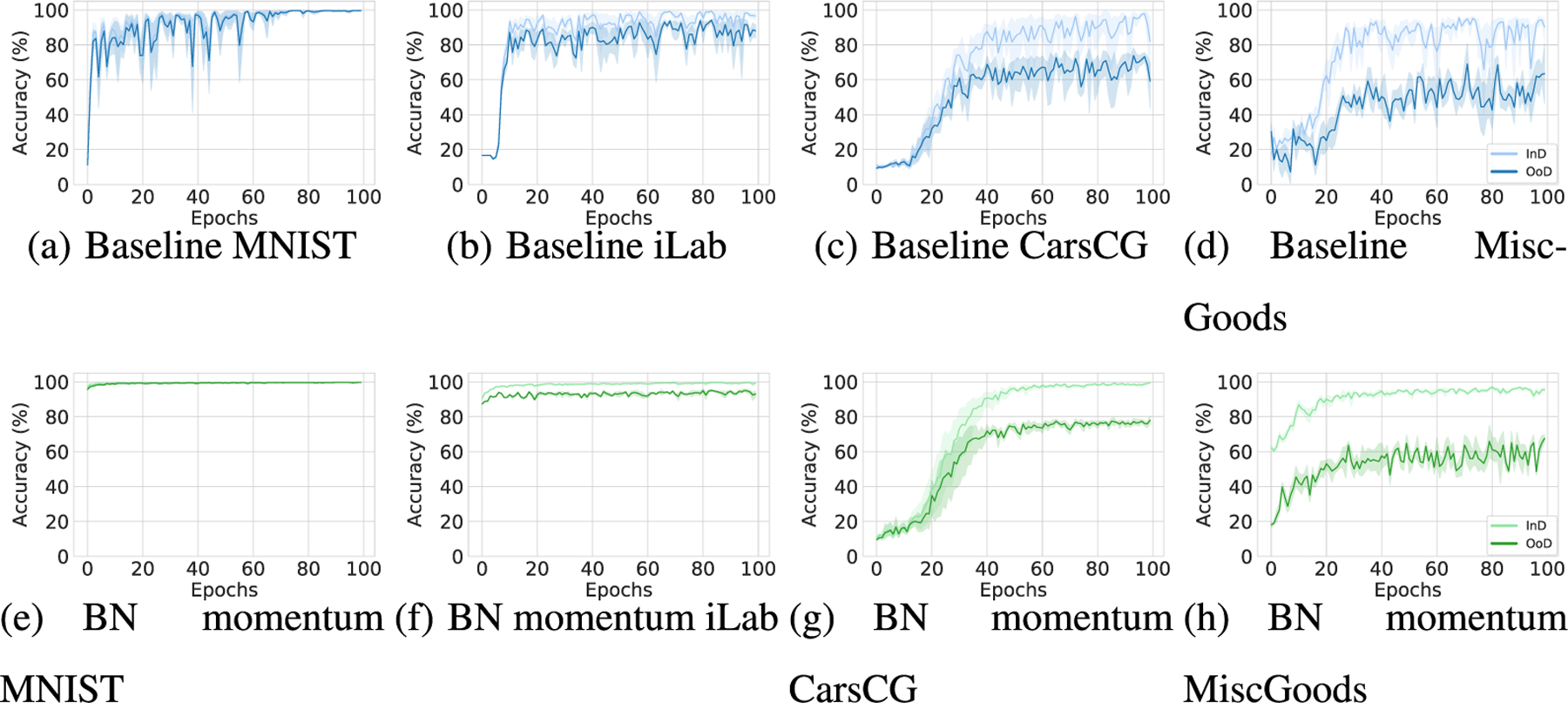
Baseline and BN momentum with high InD data diversity.

**Fig. E.7.**
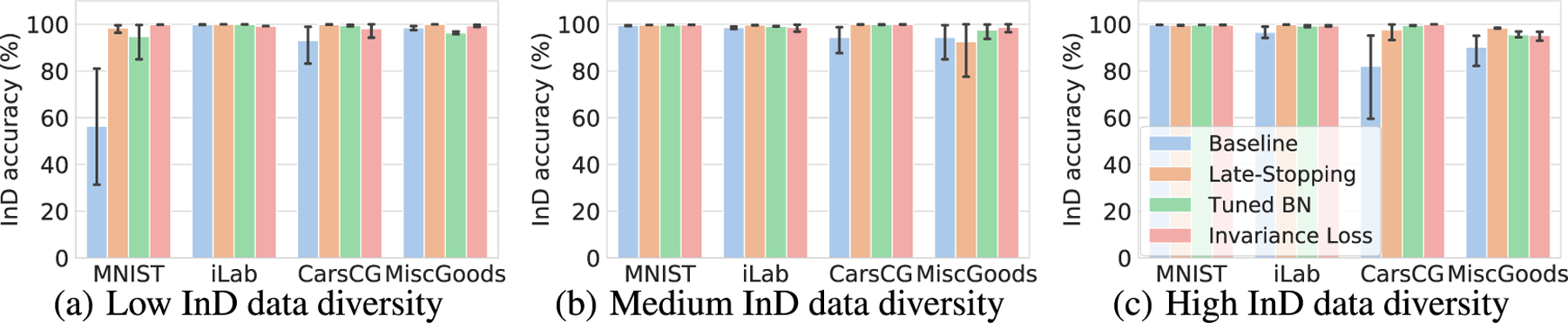
Performance improvement in mean InD accuracy.

**Fig. E.8.**
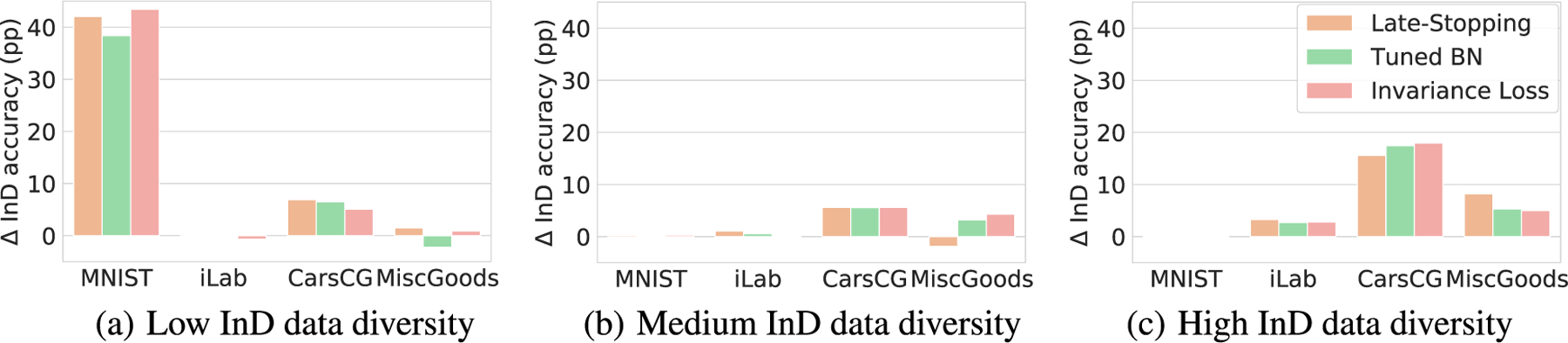
Performance improvement of difference from Baseline in mean InD accuracy.

## Data Availability

The source code used in this study is publicly available in the following GitHub repository: https://github.com/FujitsuResearch/three-approaches-ood. All datasets used in this study are publicly available (see Appendix B).

## Appendix A. Formulation of bias-controlled experiments

In bias-controlled experiment, we prepare the InD dataset ${𝒟}^{\left(\text{InD}\right)}$ that consists of images belonging to certain category (𝒞) and orientation or illuminations condition (𝒩); we denote the complement set of ${𝒟}^{\left(\text{InD}\right)}$ as ${𝒟}^{\left(\text{OoD}\right)}$. Let $x^{\left(k\right)}$ be an image and $y^{\left(k\right)}=\left(c^{\left(k\right)},n^{\left(k\right)}\right)$ be the corresponding label. We divide our dataset as follows. First, we select certain combinations of category and condition ℐ⊂𝒞×𝒩. InD dataset is sampled as

(A.1)
$${𝒟}^{\left(\text{InD}\right)}=\left\{\left(x^{\left(k\right)},y^{\left(k\right)}\right)|y^{\left(k\right)}\in ℐ\right\},$$

while the sampling process to make the combination ℐ is forced to satisfy the conditions described below. Let us define ${\left.ℐ\right|}_{𝒞}$ and ${\left.ℐ\right|}_{𝒩}$ as follows:

(A.2)
$${\left.ℐ\right|}_{𝒞}=\left\{c\in 𝒞|\left(c,n\right)\in ℐ\right\},$$

(A.3)
$${\left.ℐ\right|}_{𝒩}=\left\{n\in 𝒩|\left(c,n\right)\in ℐ\right\}.$$

We impose conditions ${\left.ℐ\right|}_{𝒞}=𝒞$ and ${\left.ℐ\right|}_{𝒩}=𝒩$ to ensure that ${𝒟}^{\left(\text{InD}\right)}$ contains all categories and all conditions like the bottom left part of Fig. 3. Training dataset ${𝒟}_{\text{train}}^{\left(\text{InD}\right)}$ is sampled from ${𝒟}^{\left(\text{InD}\right)}$ so that each combination of category and condition has the same number of images. Validation dataset ${𝒟}_{\text{val}}^{\left(\text{InD}\right)}$ is sampled from the rest of ${𝒟}^{\left(\text{InD}\right)}$ in the same way.

The OoD dataset is defined as

(A.4)
$${𝒟}^{\left(\text{OoD}\right)}≔\left\{\left(x,y\right)|y\in \left(𝒞\times 𝒩\right)\setminus ℐ\right\}.$$

InD data is sampled from ${𝒟}^{\left(\text{InD}\right)}$ so that each combination of category and condition has the same number of images.

We also define (InD) data diversity as #(ℐ)/#(𝒞×𝒩). To examine the dependency of the generalization on data diversity, we increase data diversity of ℐ by creating a set ${ℐ}^{\prime }$ with data diversity $\#\left({ℐ}^{\prime }\right)/\#(𝒞\times 𝒩)$, so that ℐ is contained in ${ℐ}^{\prime }$, *i.e*.,

(A.5)
$$ℐ\subset {ℐ}^{\prime },$$

while keeping the same amount of training examples, *i.e*.,

(A.6)
$$\#\left({𝒟}^{\left(\text{InD}\right)}\left(ℐ\right)\right)=\#\left({𝒟}^{\left(\text{InD}\right)}\left({ℐ}^{\prime }\right)\right).$$

For each z∈𝒩, we define ${\left.ℐ\right|}_{𝒞}(z)\subset 𝒞$ as follows:

(A.7)
$${\left.ℐ\right|}_{𝒞}(z)=\{c\in 𝒞|(c,z)\in ℐ\}.$$

Also, for each z∈𝒞, we define ${\left.ℐ\right|}_{𝒩}(z)\subset 𝒩$:

(A.8)
$${\left.ℐ\right|}_{𝒩}(z)=\{n\in 𝒩|(z,n)\in 𝒩\}.$$

In our experiments, we only treat the cases where

(A.9)
#(𝒞)=#(𝒩)

and keep the following additional condition to balance the combinations:

(A.10)
$$\#\left({\left.ℐ\right|}_{𝒩}\left(z\right)\right)=\#\left({\left.ℐ\right|}_{𝒩}\left(z^{\prime }\right)\right)=\#\left({\left.ℐ\right|}_{𝒞}\left(z\right)\right)=\#\left({\left.ℐ\right|}_{𝒞}\left(z^{\prime }\right)\right),\text{for}\;\text{all}\;z\;\text{and}\;z^{\prime }.$$

## Appendix B. Details of datasets

### B.1. MNIST-Positions

Starting with the MNIST dataset (LeCun et al., 1998), which is available at http://yann.lecun.com/exdb/mnist/ (Last access: Dec. 15, 2021) under the CC0 license, we created a dataset of 42 × 42 pixels with nine numbers (0 to 8) by resizing images to 14 × 14 and placing them in one of 9 possible positions in a 3 × 3 empty grid. We call it MNIST-Positions. Fig. B.1 shows the all categories and positions of MNIST-Positions. In our experiments, the numbers are considered to be the object category set 𝒞 and the positions where the numbers are placed is considered as the condition set 𝒩. Thus, it is written as #(𝒞)=#(𝒩)=9.

### B.2. iLab-Orientations

iLab-2M is a dataset created from iLab-20M dataset (Borji et al., 2016): freely and publicly available at https://bmobear.github.io/projects/viva/ (Last access: Dec. 15, 2021). The dataset consists of images of 15 categories of physical toy vehicles photographed in various orientations, elevations, lighting conditions, camera focus settings and backgrounds. It has 1.2M training images, 270k validation images, 270k test images, and each image is 256 × 256 pixels. We chose from the original iLab-2M dataset six categories – bus, car, helicopter, monster truck, plane, and tank as 𝒞 and six orientations as 𝒩. We call it iLab-Orientations. Fig. B.2 shows samples of the all categories and orientations of iLab-Orientations dataset.

### B.3. CarsCG-Orientations

CarsCG-Orientations is a new dataset that consists of images of ten models of cars in various conditions rendered by Unreal Engine version 4.25.3; this dataset is publicly available at http://dataset.jp.fujitsu.com/data/carscg/index.html. The conditions consist of ten orientations, three elevations, ten body colors, five locations and three time slots. Fig. B.3 shows the all car models (categories) and orientations (conditions) in the grid form. The details of these are as follows.

- Categories: CarsCG-Orientations dataset consists of images of the following cars – Nissan Rouge^®^, Volkswagen^®^ Golf, Volkswagen^®^ Beetle, Honda Odyssey^®^, Toyota Prius^®^, Mercedes Benz^®^ A-Class, Lexus^®^ LS, Mercedes Benz^®^ E-Class, Toyota Yaris^®^ and Volvo^®^ V40 (See Fig. B.4). We used the whole car models as categories 𝒞. Therefore the number of categories is #(𝒞)=10 in the experiments conducted in this study.
- Orientations: During the rendering process, the virtual camera (camera actor) was rotated around the yaw axis of each car from 0 to 324 degrees in units of 36 degrees. Therefore, each car model appears in the images with ten different azimuth orientations. All orientations are shown in Fig. B.5. We used the whole orientations as conditions 𝒩. Thus the number of the conditions is #(𝒩)=10 in the experiments conducted in this study.

To create variety of samples for each combination of the categories (car models) and conditions (orientations), we added other conditions as follows.

- Elevations: The virtual camera was located at three elevation angles, namely, 10, 15, and 30 degrees, during the rendering process. Sample images taken from each angle are shown in Fig. B.6.
- Body colors: Each car model is rendered with ten colors, namely, black, light blue, green, red, white, beige, dark blue, orange, plum, and silver by using Automotive Materials (a library for Unreal Engine). Fig. B.7 shows sample images of Nissan Rouge rendered with these colors.
- Locations: We used a sample environment of an urban park contained in City Park Environment Collection. We chose five locations from the sample environment and modified them for our experiments. Sample images taken at each location are shown in Fig. B.8.
- Time slots: We used Ultra Dynamic Sky 3D model set to synthesize the three different times slots, namely, daytime, twilight, and night. Fig. B.9 shows the samples of these three time slots.

The number of images and the image size are as follows.

- Number of images and image size: The total number of images of this dataset is 45k = 10 (categories) × 10 (orientations) × 3 (elevations) × 10 (body colors) × 5 (locations) × 3 (time slots). The images are rendered in 3840 × 2160 pixels and then resized to 1920 × 1080 pixels for the sake of anti-aliasing.

#### B.4. MiscGoods-Illuminations

MiscGoods-Illuminations is a subset of DAISO-10, a novel dataset constructed for this study; this dataset is publicly available at http://dataset.jp.fujitsu.com/data/daiso10/index.html. The dataset consists of images of ten physical miscellaneous goods taken with five illumination conditions, two ways of object placement, twenty object orientations, five camera angles. Images were taken with a robot arm (Fig. B.10). Fig. B.11 shows the all miscellaneous goods (categories) and illumination conditions in the grid form. The details of these are as follows.

- Categories: As shown in Fig. B.11, DAISO-10 has ten types of miscellaneous goods – stuffed dolphin, stuffed whale, metal basket, imitation plant, cup, cleaning brush, winding tape, lace yarn, bottled imitation tomatoes, and bottled imitation green apples. In this study, we selected the following five miscellaneous goods from DAISO-10 as the categories 𝒞 – stuffed dolphin, stuffed whale, metal basket, imitation plant and cup. Therefore the number of categories is #(𝒞)=5 in the experiments conducted in this study.
- Illumination conditions: As the conditions, we created five illumination conditions (lighting conditions); one is created with ceiling lights, and the rest are with a colored spotlight. All illumination conditions are shown in Fig. B.11. For spotlight conditions, the light source (PIXEL G1S^™^ RGB Video Light) was placed 23 cm in front of the object (See Fig. B.10). The parameters of the light source were H217/S141 = 8500k (white light), H0/S100 (red light), H120/S100 (green light), and H240/S100 (blue light). These parameters were set so that the condition of the illumination makes a sufficient difference in the learning experiments. We used whole illumination conditions 𝒩. Thus the number of the conditions is #(𝒩)=5 in the experiments conducted in this study.

As we did for CarCGs-Orientations, we added other conditions to create variety of samples for each combination of the categories and illumination conditions as follows.

- Object poses (ways of object placement and orientations): In this dataset, we placed each object in two representative ways of object placement for each lighting condition. Fig. B.12 shows the two ways of object placement of all objects. For additional diversity, we rotated the object every 18 degrees from 0 to 342 degrees (Fig. B.13). In total, there are 40 patterns in object pose conditions.
- Camera angles: To capture the images automatically, we created a robotic image capture system (see Fig. B.10). A camera (Intel^®^ Realsense D435) was attached to a robot arm (COBOTTA^®^), and the system captured images from five camera angles for each lighting and object pose condition (Fig. B.14). The postures were defined so that the acquired image shows the entire object pose. The series of operations from robot control to image acquisition is automated by utilizing ROS kinetic.

The number of images and the image size are as follows.

- Number of images and image size: The number of images of whole dataset is 10k = 10 (categories) × 5 (illuminations) × 2 (ways of object placement) × 20 (orientations) × 5 (camera angles), and each image size is 640 × 480 pixels.

## Appendix C. Sensitivity to hyper-parameter

In this appendix, we investigate the hyper-parameter sensitivity of the tuning batch normalization and invariance loss; for late-stopping, the epoch size is simply set to 1000 epochs. We examine hyper-parameter dependence by examining the difference between the best and second best OoD accuracy in the tuning dataset (Table C.1). We can consider the sensitivity to hyper-parameters to be small if the difference is small. The results showed that the difference was generally within 3%, although there were outliers in the low data diversity of the MNIST dataset for invariance loss and in the MiscGoods medium data diversity of the tuning dataset. Therefore, we consider the sensitivity to the hyper-parameters to be within a reasonable range.

**Table C.1:** Differences of the best and second-best OoD accuracy on the tuning dataset. This Table shows the difference between the best and second best OoD accuracy (the best OoD accuracy is shown in Parentheses) in the tuning dataset of Tuning Batch Normalization and Invariance Loss; Late-stopping is not shown because it only continues learning until 1000 epochs.

Approach	Data diversity	MNIST	iLab	CarsCG	MiscGoods	Mean
Tuned BN (%)	Low	4.96 (17.79)	0.37 (65.11)	0.06 (32.24)	0.59 (32.31)	1.50
Invariance loss (%)		15.00 (47.42)	0.54 (69.79)	0.55 (38.93)	4.21 (46.31)	5.08
Tuned BN (%)	Medium	1.08 (87.35)	0.11 (78.61)	1.60 (60.78)	7.42 (57.58)	2.55
Invariance loss (%)		0.06 (99.16)	0.18 (80.45)	0.58 (63.26)	1.07 (74.00)	0.47
Tuned BN (%)	High	0.02 (99.69)	0.14 (93.05)	4.00 (85.01)	0.27 (67.52)	1.11
Invariance loss (%)		0.05 (99.81)	0.75 (95.43)	4.14 (92.39)	1.13 (81.48)	1.52

## Appendix D. Details of experiments

ResNet-18 (He et al., 2016) is adopted as the network for all experiments. The source codes are implemented based on Python v3.6.9, using TensorFlow v2.5.0 and NumPy v1.19.5. The whole network architecture is shown in Figs. D.1 and D.2. All neurons employ the rectified linear function g(z)=max{0,z} and satisfy $a^{nm}(x)\geq 0$. Glorot uniform initializer (Glorot & Bengio, 2010) is adopted for the network weights initialization for all experiments. We use BatchNormalization to standardize the inputs to a layer for each mini-batch. We use it for stabilizing the learning process and reducing the number of training epochs. We do not use any data augmentations. Invariance loss is applied to the last fully-connected layer “activation_17” with 512 neurons shown in Fig. D.1. Adam (Kingma & Ba, 2015) is employed as the optimization algorithm. The cross-entropy loss is employed as the loss L. The pixels of images are normalized within 0 to 1 as a preprocessing for all datasets. The epoch size and batch size are confirmed to produce reasonable accuracy in the baseline case for each dataset and we employ the same values for all experiments with the same dataset. For example, we use 100 and 256 as epoch size and batch size, respectively, for MNIST-Positions. The values of hyper-parameters are summarized in Table D.1.

**Table D.1:** Hyper-parameters used for each dataset.

Dataset	Epoch size	Preprocessing	Weights initialization	Batch size
MNIST-Positions	100	Divide by 255	Glorot uniform initializer	256
iLab-Orientations	100	Divide by 255	Glorot uniform initializer	256
CarsCG-Orientations	100	Divide by 255	Glorot uniform initializer	32
MiscGoods-Illuminations	100	Divide by 255	Glorot uniform initializer	32

We have employed four Tesla V100 GPUs for the experiments. The preparation of training dataset ${𝒟}_{\text{train}}^{\left(\text{InD}\right)}$, InD validation dataset ${𝒟}_{\text{val}}^{\left(\text{InD}\right)}$, and OoD dataset ${𝒟}^{\left(\text{OoD}\right)}$ has been conducted as follows.

- MNIST-Positions: We use images of the original MNIST-Positions with image size of 42 × 42 pixels. InD dataset and OoD dataset are prepared in the way described in Section 3.1. The number of train dataset is $\#\left({𝒟}_{\text{train}}^{\left(\text{InD}\right)}\right)=54000$. We use $\#\left({𝒟}_{\text{val}}^{\left(\text{InD}\right)}\right)=8000$ for InD validation dataset. The number of OoD dataset is $\#\left({𝒟}^{\left(\text{OoD}\right)}\right)=8000$.
- iLab-Orientations: We resize the images to 64 × 64 pixels. InD dataset and OoD dataset are prepared in the way described in Section 3.1. The number of train dataset is $\#\left({𝒟}_{\text{train}}^{\left(\text{InD}\right)}\right)=18000$. We use $\#\left({𝒟}_{\text{val}}^{\left(\text{InD}\right)}\right)=8000$ for InD validation dataset. The number of OoD dataset is $\#\left({𝒟}^{\left(OoD\right)}\right)=8000$.
- CarsCG-Orientations: We resize the images to 224 × 224 pixels. InD dataset and OoD dataset are prepared in the way described in Section 3.1. The number of train dataset is $\#\left({𝒟}_{\text{train}}^{\left(\text{InD}\right)}\right)=3400$. We use $\#\left({𝒟}_{\text{val}}^{\left(\text{InD}\right)}\right)=450$ for InD validation dataset. The number of OoD dataset is $\#\left({𝒟}^{\left(OoD\right)}\right)=800$.
- MiscGoods-Illuminations: We resize the images to 224 × 224 pixels. InD dataset and OoD dataset are prepared in the way described in Section 3.1. The number of train dataset is $\#\left({𝒟}_{\text{train}}^{\left(\text{InD}\right)}\right)=800$. We use $\#\left({𝒟}_{\text{val}}^{\left(\text{InD}\right)}\right)=200$ for InD validation dataset. The number of OoD dataset is $\#\left({𝒟}^{\left(\text{OoD}\right)}\right)=400$.

## Appendix E. Additional results of experiments

InD accuracy and OoD accuracy learning curves with all dataset and all diversity corresponding to Fig. 1(a) are available in Figs. E.1 E.2 E.3. Furthermore InD accuracy and OoD accuracy learning curves with all dataset and all diversity corresponding to Fig. 1(b) are available in Figs. E.4 E.5 E.6. InD accuracy corresponding to Figs. 5(a) 5(b) 5(c) is available in Fig. E.7. InD accuracy of difference from baseline corresponding to Figs. 5(d) 5(e) 5(f) is also available in Fig. E.8. The experiments for measuring accuracy are exactly same as what we reported in the main body of the paper.

## Appendix F. Details of combined method

For the best of three approaches, we choose the one with the highest OoD accuracy calculated on the dataset for hyper-parameter tuning among late stopping, tuning batch normalization momentum, and invariance loss. The combined three approaches employ the hyper-parameters that determined in Section 6: epoch size for longer epochs, momentum parameter β for tuning batch normalization momentum, and learning rate, pairing interval and the value λ for invariance loss. If the InD accuracy drops to a chance, the learning rate is multiplied by 0.1 and the training is re-started.

## Appendix G. Comparison with the state-of-the-art methods

We now compare the existing state-of-the-art OoD algorithms with the three approaches we introduce. We employ PGI (Ahmed et al., 2021) and CMMD (Li et al., 2018) implemented in Ahmed et al. (2021) as existing state-of-the-art algorithms.^1^ For consistency with previous works (Ahmed et al., 2021), we use Wide-ResNet (Zagoruyko & Komodakis, 2016) for the network and CarsCG for the dataset. Also, the images are scaled down to 64 × 64, and the data is augmented with slides and inversions. Hyper-parameters are optimized in the same way as in the main text for the three approaches. For PGI and CMMD, we perform a grid search using tuning dataset in learning rate in {0.1, 0.01, 0.001, 0.0001}, portion weight for EIIL (Creager et al., 2021) in {10, 1, 0.1, 0.01, 0.001}, and weight for loss term in {1, 0.1, 0.01, 0.001, 0.0001}; this is the reasonable subset of grid search as in Ahmed et al. (2021). For other parameters, we fully employ the parameters used in Ahmed et al. (2021). We perform five trials on the test dataset using the selected hyper-parameters. Table G.1 shows the median, and the minimum and maximum in parenthesis, of the results. Existing state-of-the-art methods improved performance from baseline with a few exceptions, but at least one of the three approaches outperform these state-of-the-art methods in all data diversity.

**Table G.1:** Comparison with the state-of-the-art methods. This table shows the OoD performance of the baseline, three approaches, PGI, and CMMD. We performed five trials for all methods and this table shows the median, and the minimum and maximum in parenthesis.

Approach	CarsCG (low)	CarsCG (medium)	CarsCG (high)
Baseline (%)	25.4 (19.8–28.3)	66.9 (40.6–71.1)	75.9 (36.5–80.5)
Late-stopping (%)	**34.0 (32.4–34.6)**	**71.5 (70.3–73.8)**	**80.9 (67.0–82.8)**
Tuned BN (%)	30.4 (26.6–33.3)	71.4 (69.9–73.1)	78.0 (72.0–83.5)
Invariance loss (%)	30.9 (28.5–33.6)	69.8 (25.5–74.1)	79.9 (71.3–84.3)
PGI (%)	24.6 (23.1–26.1)	68.0 (15.0–71.6)	75.3 (71.8–78.1)
CMMD (%)	31.3 (30.0–33.0)	64.0 (32.6–69.5)	79.5 (36.1–80.6)

Finally, we also explore combining PGI and CMMD with tuning batch normalization and late-stopping. Table G.2 shows the results. In all cases, performance improvement up to +8.7 are observed when the existing state-of-the-art methods (PGI and CMMD) are combined with our approaches. This shows that our three approaches tackle complementary aspects from state-of-the-art methods, and are versatile and easy to be effectively combined with previous works.

**Table G.2:** Performance improvement of the state-of-the-art methods by tuning batch normalization and late-stopping. This table shows the OoD performance improvement (Δ) of the combination of the state-of-the-art methods with tuning batch normalization and late-stopping (denoted by co). We performed five trials for all methods and this table shows the median (max-min).

Approach	CarsCG (low)	Δ	CarsCG (medium)	Δ	CarsCG (high)	Δ
Invariance loss (%)	30.9 (28.5–33.6)		69.8 (25.5–74.1)		79.9 (71.3–84.3)	
Invariance loss (co) (%)	**34.9 (33.6–37.6)**	+4.0	67.1 (60.9–72.3)	−2.7	**84.0 (80.8–86.1)**	+4.1
PGI (%)	24.6 (23.1–26.1)		68.0 (15.0–71.6)		75.3 (71.8–78.1)	
PGI (co) (%)	33.3 (31.1–35.6)	+8.7	**72.5 (68.4–73.1)**	+4.5	83.9 (81.5–86.1)	+8.6
CMMD (%)	31.3 (30.0–33.0)		64.0 (32.6–69.5)		79.5 (36.1–80.6)	
CMMD (co) (%)	34.5 (32.4–37.8)	+3.2	70.9 (68.9–72.3)	+6.9	81.1 (14.9–88.1)	+1.6

## Appendix H. Visualization of the latent space

In this appendix, we visualize the latent spaces obtained by the baseline method and three approaches. Table H.1 shows the results of applying t-SNE to the latent space of the last fully connected layer of ResNet-18 trained by the CarsCG dataset. Three approaches are confirmed to increase cluster concentration. This result is consistent with the improvement of the SI score by three approaches shown in Section 6.3.

**Table H.1:** Latent space visualization using t-SNE. Each cell of this table shows the t-SNE visualization of the activity of the latent layer of the network that each approach has been applied (row), trained on each data diversity (column). Each color expresses a car model.

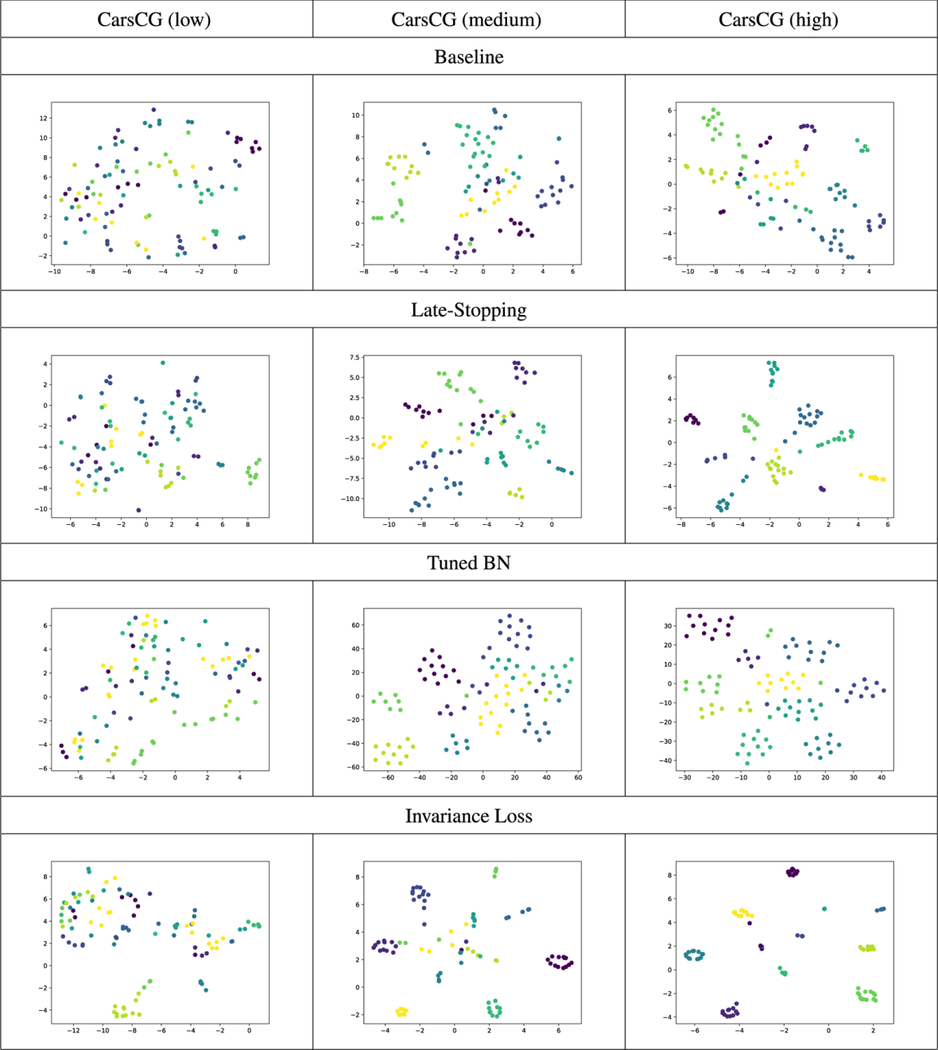

## Appendix I. Abbreviation list

- OoD: out-of-distribution
- InD: in-distribution
- DNN: deep neural network
- BN: batch normalization
- MNIST dataset: modified national institute of standards and technology dataset
- CarsCG dataset: cars computer graphics dataset
- MiscGoods dataset: miscellaneous goods dataset
- 3D: three dimension
- EIIL: environment inference for invariant learning
- SI score: selectivity and invariance score
- ResNet: residual network
- ReLU: rectified linear unit
- CC: creative commons
